# Synergy between intrinsically disordered domains and structured proteins amplifies membrane curvature sensing

**DOI:** 10.1038/s41467-018-06532-3

**Published:** 2018-10-08

**Authors:** Wade F. Zeno, Upayan Baul, Wilton T. Snead, Andre C. M. DeGroot, Liping Wang, Eileen M. Lafer, D. Thirumalai, Jeanne C. Stachowiak

**Affiliations:** 1 Department of Biomedical Engineering, The University of Texas at Austin, Austin, TX 78712 USA; 2 Department of Chemistry, The University of Texas at Austin, Austin, TX 78712 USA; 3 Department of Biochemistry and Structural Biology, The University of Texas Health Science Center at San Antonio, San Antonio, TX 78229 USA; 4 Institute for Cellular and Molecular Biology, The University of Texas at Austin, Austin, TX 78712 USA

## Abstract

The ability of proteins to sense membrane curvature is essential to cellular function. All known sensing mechanisms rely on protein domains with specific structural features such as wedge-like amphipathic helices and crescent-shaped BAR domains. Yet many proteins that contain these domains also contain large intrinsically disordered regions. Here we report that disordered domains are themselves potent sensors of membrane curvature. Comparison of Monte Carlo simulations with in vitro and live-cell measurements demonstrates that the polymer-like behavior of disordered domains found in endocytic proteins drives them to partition preferentially to convex membrane surfaces, which place fewer geometric constraints on their conformational entropy. Further, proteins containing both structured curvature sensors and disordered regions are more than twice as curvature sensitive as their respective structured domains alone. These findings demonstrate an entropic mechanism of curvature sensing that is independent of protein structure and illustrate how structured and disordered domains can synergistically enhance curvature sensitivity.

## Abstract (web summary)

Many proteins which sense membrane curvature contain intrinsically disordered domains. Here the authors use Monte Carlo simulations combined with experimental approaches and report that disordered domains are potent sensors of membrane curvature.

## Introduction

Curved membrane structures such as endocytic pits, viral buds, filopodia, and tubular organelles are essential to cellular physiology^1^. Formation of these structures requires that proteins with the capacity to generate membrane curvature assemble together at specific locations on cellular membrane surfaces^2,3^. These proteins are thought to sense the curvature of the surrounding membrane and bind preferentially to curved sites, progressively enhancing membrane curvature. Two primary mechanisms of curvature sensing have been well-characterized: (i) membrane scaffolding by crescent-shaped BAR (Bin/Amphiphysin/Rvs) domains^4^, which match their curvature to that of the membrane, and (ii) detection of membrane defects by amphipathic helices^5^, which insert like wedges between the head groups of lipids found in highly curved membrane surfaces. Both of these mechanisms rely on specific protein structural features. However, these structured domains frequently constitute only a small fraction of the mass of the protein molecules that contain them. Specifically, large intrinsically disordered protein (IDP) domains, which lack well-defined secondary structures^6^, are often also present within the same protein molecules. Several examples of multi-domain proteins that couple structured curvature sensors with substantial regions of intrinsic disorder are found in the clathrin-mediated endocytic pathway. Specifically, Epsin1 consists of an ENTH (Epsin N-terminal homology^7^) domain, which contains a curvature-sensing amphipathic helix, followed by a disordered domain of more than 400 amino acids^8^. Similarly, AP180 consists of a curvature sensing ANTH (AP180 N-terminal homology^9^) domain, followed by an intrinsically disordered domain of more than 500 amino acids^8,10^. BAR domains are also frequently found in combination with substantial disordered regions. For example, Amphiphysin1 contains a crescent-shaped N-BAR (N-terminal BAR) domain^4^ but also contains a substantial disordered region of nearly 400 amino acids^11^.

In studies of curvature sensing, structured domains have nearly always been studied in isolation from disordered regions based on the assumption that a well-defined structure is required for curvature sensing. However, the polymer-like behavior of many well-solvated IDP domains^12–14^ suggests a potential role in curvature sensing. Specifically, tethering a polymer-like molecule to a surface is known to significantly reduce its entropy by restricting the number of conformations available to it^13–15^. However, this loss can in principle be partially recovered by tethering polymers to convex surfaces, which curve toward them. Here the entropy of the polymer chain would be expected to increase as the convex curvature of the substrate increases, potentially driving partitioning of tethered polymers to more highly curved substrates. Entropic mechanisms of curvature sensing have not been explored to date. However, it is increasingly recognized that intrinsically disordered domains are integral components of many proteins involved in membrane remodeling and coated-vesicle biogenesis^11,16,17^. Motivated by this reasoning, here we ask whether membrane-bound IDPs can sense membrane curvature using entropic mechanisms.

To investigate the ability of disordered domains to sense membrane curvature, we measure membrane binding as a function of vesicle diameter (20–200 nm) for the disordered domains of AP180, Epsin1, and Amiphiphysin1. In each case we find a substantial increase in binding as membrane curvature increases, the level of which is comparable to the established structure-based curvature sensors, ENTH and N-BAR. Further, curvature sensing by IDP domains decreases with increasing rigidity of the peptide chain, in agreement with Monte Carlo simulations that capture the impact of substrate curvature on chain entropy. Further, we investigate the ability of IDP domains to sense membrane curvature when displayed on the plasma membrane of live mammalian cells. By analyzing the differential partitioning of these domains between highly curved filopodia and the relatively flat plasma membrane, we find that IDPs exhibit increased partitioning to the convex outer filopodial surface and reduced partitioning to the concave inner surface. Finally, examining full-length endocytic proteins reveals that disordered and structured curvature sensing domains present in the same protein work together synergistically, more than doubling the curvature sensitivity of any individual domain, structured or disordered. Taken together this work demonstrates that IDP domains are a critical, yet previously unknown, class of curvature sensors. These findings fundamentally alter our understanding of how proteins sense membrane curvature by demonstrating a previously unknown entropic mechanism of curvature sensing that can substantially amplify the performance of previously identified structured curvature sensors. Therefore, this work necessitates a substantial expansion and reexamination of the set of proteins responsible for sensing the curvature of membrane structures in diverse biological processes.

## Results

### The disordered domain of AP180 senses membrane curvature

AP180, an adaptor protein in the clathrin-mediated endocytic pathway, is one of several proteins responsible for assembly of the clathrin coat during vesicle biogenesis^18–20^. AP180’s intrinsically disordered C-terminal domain lacks substantial regions of secondary structure and is highly water soluble^8,10,21^. The disordered structure of AP180’s C-terminal domain has been verified using multiple experimental techniques, including circular dichroism spectroscopy^8^, NMR spectroscopy^10^, fluorescence correlation spectroscopy^21^, gel filtration^8^, and analytical ultracentrifugation^8^. Additionally, no significant secondary structure in this domain, or other disordered domains considered in this work, was predicted by computational sequence analysis (Supplementary Figs. 1–3). Therefore, we used the intrinsically disordered C-terminal domain of AP180 (Rat AP180, residues 328–896) as a model protein for evaluating the ability of IDP domains to sense membrane curvature. To attach this domain to membrane vesicles containing Ni-NTA DOGS lipids, we added an N-terminal hexa-histidine tag to AP180’s C-terminal domain (his-AP180CTD). The histidine-Ni interaction ensures that the disordered domains bind to the membrane surfaces at their N-termini^22^. Importantly, his-AP180CTD did not bind measurably to membranes  lacking DGS-NTA lipids (Supplementary Fig. 4a-c), indicating that other residues or motifs within the disordered protein did not interact significantly with the membrane. As a positive control for curvature sensing, we examined the wild-type Epsin N-terminal Homology domain (wt-ENTH), which is known to sense membrane curvature by inserting its N-terminal amphipathic helix (residues 1–16) into lipid packing defects present in highly curved membrane surfaces^23^. As a negative control, we removed ENTH’s curvature sensing helix and replaced it with a hexa-histidine tag (his-ΔENTH).

**Fig. 1:**
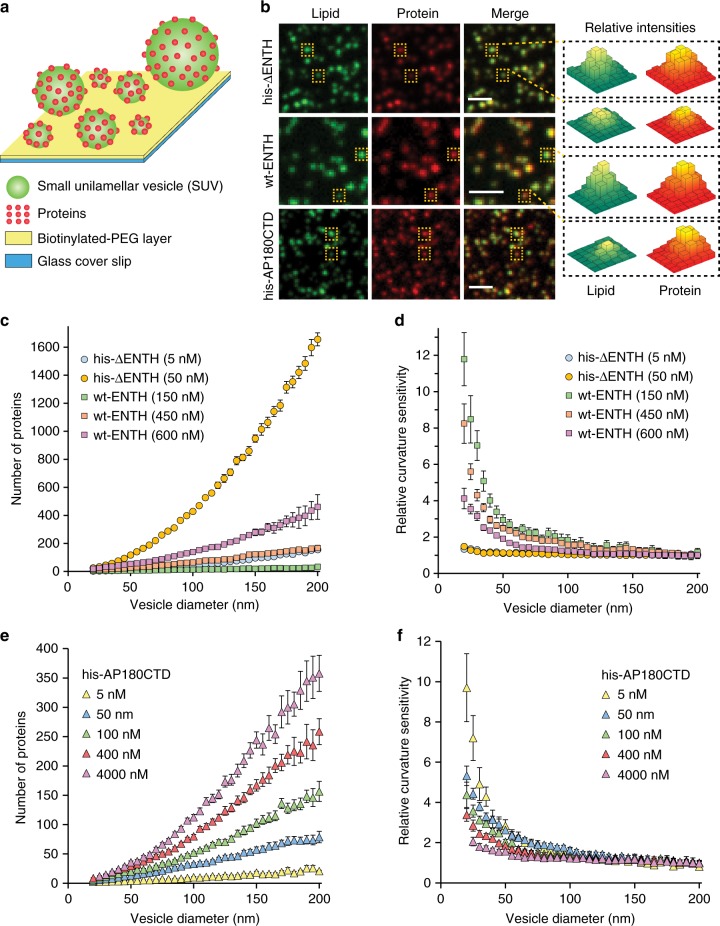
The disordered C-terminal domain of AP180 senses membrane curvature. **a** Schematic of the curvature sensing assay (see text). **b** Lipid, protein, and merged fluorescent images of SUVs (30 nm extruded) and membrane-bound proteins. Yellow boxes highlight SUVs of varying brightness, corresponding to varying diameter. In the presence of wt-ENTH or his-AP180CTD, protein (red) to lipid (green) fluorescence ratio appears higher for smaller (less bright in lipid channel) SUVs. Vesicles used for his-ΔENTH and his-AP180CTD binding contained 80% DOPC, 16% DGS-NTA, 2% DP-EG10-biotin, and 2% Oregon Green-DHPE. SUVs used for wt-ENTH binding contained 88.5% DOPC, 7.5% PI(4,5)P2, 2% DP-EG10-biotin, and 2% Oregon Green-DHPE. All proteins were labeled with ATTO-594 dye. Scale bar represents 2 μm. **c** Number of proteins bound as a function of SUV diameter for his-ΔENTH and wt-ENTH. **d** Relative curvature sensitivities of his-ΔENTH and wt-ENTH. **e** Number of proteins bound as a function of SUV diameter for his-AP180CTD. **f** Relative curvature sensitivity for his-AP180CTD. All data in (**c**–**f**) is presented as a 5 nm-increment moving average of the corresponding raw data (>1000 data points), with error bars represented by the 95% confidence interval of the mean. Each bin in (**c**–**f**) contains *N* = 24–369 data points acquired cumulatively from three independent replicates

A tethered vesicle assay, depicted in Fig. 1a, was used to measure the curvature sensing abilities of these proteins. Small unilamellar vesicles (SUVs) with diameters varying from 20 to 200 nm were tethered to a coverslip surface as described previously^5^ (see 'Methods'). This range of vesicle diameters encompasses most curved biological membrane structures^2^. Protein containing solutions were then added to the tethered vesicles and binding of proteins to vesicle surfaces was observed using confocal fluorescence microscopy. SUVs contained the fluorophore Oregon Green 488 1,2-dihexadecanoyl-sn-glycero-3-phosphoethanolamine (Oregon Green-DHPE), while proteins were labelled with ATTO-594, conjugated to lysine residues within each protein (see 'Methods'). Binding of his-ΔENTH, wt-ENTH, and his-AP180CTD to immobilized SUVs is depicted in Fig. 1b. For tethered vesicles exposed to his-ΔENTH, the ratio of fluorescence intensities in the protein and lipid fluorescence channels was approximately constant across the range of SUV diameter, such that the SUVs appeared to have a uniform yellow color when the lipid (green) and protein (red) channels were merged (Fig. 1b, top row). This constant ratio indicates a lack of curvature sensitivity, which is expected since the curvature sensing amphipathic helix is absent in his-ΔENTH. In contrast, for wt-ENTH, brighter puncta in the lipid channel, which correspond to vesicles of larger diameter, had low protein to lipid channel intensity ratios, causing them to appear greener in merged images. Conversely, dimmer puncta in the lipid channel, which correspond to vesicles of smaller diameter, had high protein to lipid ratios, causing them to appear redder in merged images (Fig. 1b, center). This distribution of intensity ratios indicates that wt-ENTH binds more strongly to more highly curved vesicles, which is expected owing to the presence of its amphipathic helix. Similarly to wt-ENTH, his-AP180CTD displayed sensitivity to membrane curvature, with protein to lipid channel intensity ratio increasing with decreasing vesicle diameter (Fig. 1b, bottom).

To quantify and compare the curvature sensitivity of these proteins, we used the fluorescent intensity of colocalized puncta in the lipid and protein fluorescent channels to estimate the number of membrane-bound proteins per SUV as a function of SUV diameter, Fig. 1c–f. We estimated SUV diameter by comparing the average Oregon Green DHPE fluorescence intensity per tethered vesicle to the average vesicle diameter obtained from dynamic light scattering, as described previously^5^ and detailed in the 'Methods' section, Supplementary Figures 5–8. We estimated the number of proteins bound to each vesicle by dividing the fluorescence intensity of each vesicle in the protein channel by the calibrated fluorescence intensity of a single dye-labeled protein as described in 'Methods', Supplementary Figures 8–9. As expected, the number of bound proteins increased monotonically with increasing vesicle diameter (Fig. 1c). Figure 1d plots relative curvature sensitivity as a function of SUV diameter. Here relative curvature sensitivity is defined as the normalized protein density, where the number of membrane-bound proteins per membrane area at each vesicle diameter is divided by the number of membrane-bound proteins per membrane area for vesicles of the reference diameter, 200 nm (i.e., density/density_diameter = 200_). The curvature sensitivity of wt-ENTH is evident in Fig. 1d, as the density of membrane-bound proteins increases by 12-fold for vesicles of 20 nm diameter in comparison to vesicles of 200 nm diameter, similar to previous observations for the wt-ENTH domain^23^ and for isolated amphipathic helices^5^. However, as protein concentration in solution increased from 150 to 600 nM, curvature sensitivity declined owing to saturation of the membrane surface, as expected^24^. In contrast, the density of membrane-bound his-ΔENTH proteins was relatively constant over all SUV diameters and protein concentrations, indicating a lack of curvature sensitivity, as expected. The lack of significant curvature sensitivity by his-ΔENTH indicates that the histidine-Ni-lipid interaction, which was also used to tether AP180CTD to membrane surfaces, does not in itself give rise to curvature sensing behavior. However, we cannot rule out the possibility that the histidine-lipid interaction may modify the local membrane entropy and thereby influence curvature sensing. Notably, protein concentrations and membrane composition were chosen to achieve comparable ranges of membrane binding for his-ΔENTH and wt-ENTH (Fig. 1 caption).

**Fig. 2:**
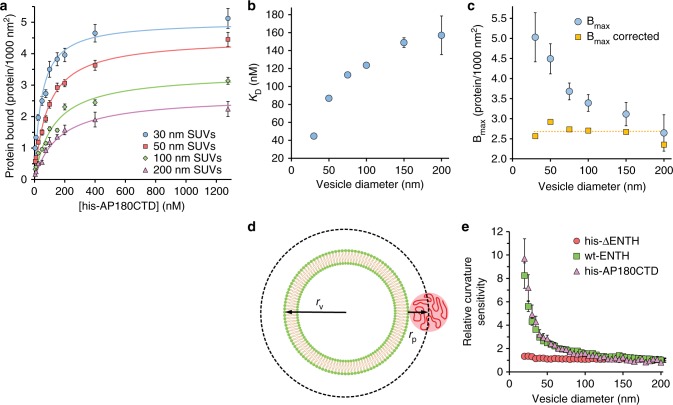
Impact of membrane curvature on IDP binding equilibria. **a** Binding curves for his-AP180CTD. **b** Regressed values for the dissociation constant (*K*_D_) as a function of SUV diameter. **c** Regressed values for maximal protein binding (*B*_max_) as a function of SUV diameter, and the corrected values after geometrical effects are removed. The dashed yellow line represents the average *B*_max_ corrected value (2.7 × 10^−3^ proteins nm^−^^2^) of the six square data points. The experimental results shown as symbols in (**a**) were fitted to Eq. 1. The resulting fit parameters are shown in (**b**, **c**). **d** Schematic of the geometric effect described by Eq. 2. **e** Curvature sensitivity comparisons of his-ΔENTH, wt-ENTH, and his-AP180CTD at approximately 3% average membrane coverage. The solution concentrations required to achieve this level of membrane coverage were 5 nM for his-ΔENTH, 450 nM for wt-ENTH, and 5 nM for his-AP180CTD. Error bars in (**a**, **e**) are represented by the 95% confidence interval of the mean. Each bin in (**a**) contains *N* = 23–338 data points acquired cumulatively from three independent replicates. Similarly, each bin in (**e**) contains *N* = 36–358 data points. Error bars in (**b**, **c**) are represented by the standard deviation of three regression values obtained from fitting the mean, upper error, and lower error bounds in (**a**)

In comparison, Fig. 1e depicts a monotonic increase in the amount of bound protein with increasing vesicle diameter for his-AP180CTD over a range of protein concentrations in solution. When SUVs were exposed to a 5 nM solution of his-AP180CTD (Fig. 1f), the density of membrane-bound proteins was approximately 10-fold higher for 20 nm diameter vesicles in comparison to vesicles with 200 nm diameter. Similar to wt-ENTH, as the concentration of protein in solution increased from 5 to 4000 nM, curvature sensitivity steadily declined. The level of curvature sensitivity observed for his-AP180CTD is therefore comparable to that observed for wt-ENTH. Additionally, we compared a C-terminally his-tagged version of AP180CTD with the N-terminally his-tagged variant used in Figs. 1 and 2 (Supplementary Fig. 10), and observed nearly identical levels of curvature sensitivity. This result demonstrates that curvature sensing is not strongly dependent on the proximity of specific residues to the point of membrane attachment, as would be expected for a mechanism that depends primarily on molecular entropy rather than protein structural motifs. In the next section we quantify the impact of membrane curvature on the binding affinity and capacity of his-AP180CTD. These results facilitate a direct comparison between the curvature sensitivity of disordered and structured curvature sensors.

### Impact of membrane curvature on IDP binding equilibria

Using the data in Fig. 1, binding curves were generated to determine the impact of membrane curvature on the affinity and capacity of AP180-CTD binding to membrane surfaces, Fig. 2a and Supplementary Figure 11. As curvature increased the apparent binding capacity appeared to saturate at increasing levels, while the apparent dissociation constant (*K*_D_), progressively decreased, as indicated by the steepening of the initial slopes. To quantify these effects, we fit a Langmuir adsorption isotherm (Eq. 1) to the binding curves,

1 $${\mathrm{Protein}}\,{\mathrm{bound = }}\frac{{B_{{\mathrm{max}}}\left[ {{\mathrm{AP180}}} \right]}}{{K_D{\mathrm{ + }}\left[ {{\mathrm{AP180}}} \right]}}$$

where *B*_max_ corresponds to the maximum of membrane-bound protein, [AP180] is the his-AP180CTD concentration in solution, and *K*_D_ is the dissociation constant. *K*_D_ increased from 40 to 160 nM as SUV diameter increased from 30 to 200 nm, indicating that AP180CTD bound more strongly to vesicles of smaller diameter (Fig. 2b). *B*_max_ decreased from 5 to 2.6 × 10^−3^ proteins nm^−^^2^ as SUV diameter increased from 30 to 200 nm (Fig. 2c). We attribute this behavior to the geometrical effect illustrated in Fig. 2d. Specifically, if bound proteins occupy an approximately spherical plane that is located a radial distance equal to the average protein radius, *r*_P_, away from the SUV surface, then the fractional difference in surface area between this plane and the plane of the membrane surface increases with decreasing SUV diameter, leading to an increased binding capacity for small SUVs, as illustrated by Eq. 2.

2 $$\frac{{A_{{\mathrm{protein}}}}}{{A_{{\mathrm{vesicle}}}}} = \frac{{\left( {r_v + r_p} \right)^2}}{{r_v^2}} = \frac{{B_{{\mathrm{max}}}}}{{B_{{\mathrm{max}}\,{\mathrm{corrected}}}}}$$

In Fig. 2c, *B*_max_ and the corresponding *B*_max corrected_ values, which were calculated from Eq. 2, are plotted. An average *B*_max corrected_ value of 2.7 × 10^−^^3^ proteins nm^−^^2^ was obtained. For these *B*_max corrected_ calculations, a value of 6.0 nm was used for *r*_p_, as measured by fluorescence correlation spectroscopy (Supplementary Figs. 12–13, Supplementary Table 1). This value of *r*_p_ is in reasonable agreement with values reported for AP180CTD^8,21^, suggesting that each AP180CTD molecule occupies approximately 115 nm^2^ on the membrane surface. Due to the polymer-like nature of IDPs, *r*_p_ is expected to vary with vesicle curvature. However, this variation is relatively small and does not alter the interpretation of the data (Supplementary Fig. 14).

The fractional protein coverage on a membrane surface is equivalent to the probability that a protein binding site is occupied. Curvature sensitivity is reduced when this probability increases, as shown in Fig. 1d, f. Therefore, comparisons of the curvature sensitivity of two different proteins must occur under conditions for which each protein covers the same fraction of the membrane surface, averaged over the population of tethered vesicles. A detailed justification of this equivalency criterion is included in the supplementary text (Supplementary Equation 4). By holding the membrane coverage constant, we effectively reduce the experimental system to a thermodynamic canonical ensemble in which the temperature, volume, and fraction of the membrane surface occupied by proteins are all held constant. Within this ensemble, curvature sensitivity is simply the relative favorable partitioning of proteins to vesicles of higher curvature. Using a membrane surface area value of 15 nm^2^ for wt-ENTH^25,26^ and 115 nm^2^ for his-AP180CTD, the relative curvature sensitivities of these two proteins were compared under conditions for which each protein covered approximately 3% of the SUV surfaces, averaged over the entire population of tethered vesicles (Fig. 2e). Here, protein coverage is defined as the total projected area of proteins (i.e., number of proteins multiplied by area of a single protein) divided by the total surface area of the vesicles. A protein coverage of 3% represents a relatively dilute regime. Therefore protein–protein interactions were neglected. Comparing the curvature sensitivity of his-AP180CTD and wt-ENTH under these conditions reveals that they achieve 10-fold and 8-fold greater binding density to 20 nm vesicles in comparison to 200 nm vesicles, respectively. These results demonstrate that the IDP domain of AP180 has a similar sensitivity to membrane curvature in comparison to wt-ENTH, an established structure-based sensor of membrane curvature.

To further demonstrate the ability of a random polymer to sense membrane curvature, we conjugated a 40 kDa polyethylene glycol (PEG) chain to histidine-tagged Green Fluorescent Protein (his-GFP). The resulting shift in molecular weight was confirmed using fluorescence correlation spectroscopy (Supplementary Fig. 15). When examined prior to conjugation with PEG, his-GFP displayed minimal curvature sensing abilities, similar to those of his-ENTH. However, following conjugation with PEG, we observed substantial curvature sensing (Supplementary Fig. 16). As a homopolymer lacking in secondary structural motifs and unable to bind to membranes directly (Supplementary Fig. 4d-f), the ability of a PEG chain to impart curvature sensing abilities to GFP further suggests an entropic mechanism of curvature sensing. Therefore, we next probed the detailed mechanism behind the ability of intrinsically disordered domains to sense membrane curvature.

### Curvature sensing arises from IDP conformational entropy

There are at least two potential explanations for the ability of IDPs to sense membrane curvature. One possible explanation is proposed in the introduction of this report—that curved substrates allow greater conformational entropy of the disordered amino acid chain of the IDP. However, a second possible explanation is that curved surfaces, which increase the volume available per IDP chain, reduce electrostatic repulsions between neighboring membrane-bound IDPs, leading to increased binding to curved membrane surfaces. Notably, the IDP domain of AP180 contains 11% charged residues and has a net charge of approximately negative 30 near neutral pH, such that chain–chain and residue–residue electrostatic interactions may be significant. Importantly, we would expect these two mechanisms, one relying on chain entropy and the other relying on chain–chain repulsion, to yield opposite outcomes as electrostatic screening increases. Specifically, screening charges on the IDP chain by increasing the salt concentration in solution would be expected to decrease residue–residue repulsion, leading to a less rigid, more compact IDP chain with larger conformational entropy. Therefore, if curvature sensitivity arises primarily from chain entropy, then increasing salt concentration would be expected to increase curvature sensitivity. In contrast, increasing salt concentration would decrease the electrostatic repulsion between chains, such that curvature sensitivity should decrease if it arises primarily from chain–chain repulsion.

**Fig. 3:**
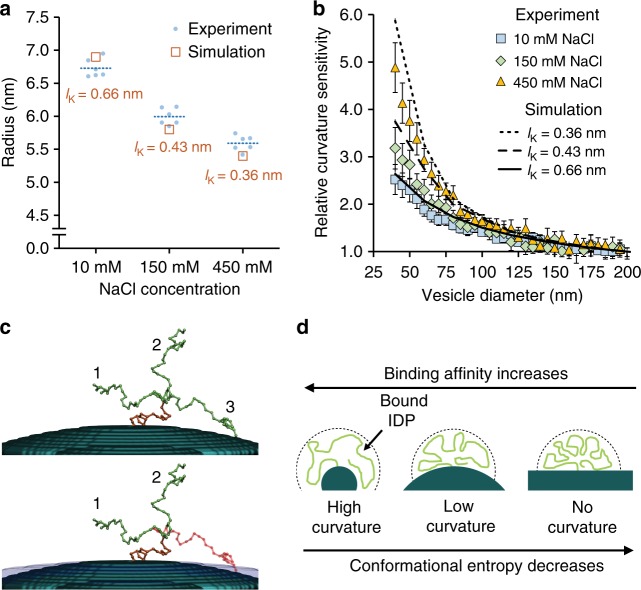
Curvature sensing arises from IDP conformational entropy. **a** Comparison of experimentally measured and simulated change in protein radius as a function of salt concentration. Dashed lines represent the average value of six experimental replicates. **b** Comparison of experimentally measured and simulated curvature sensitivity as a function of salt concentration, Kuhn length, *l*_K_. Relative sensitivities are normalized to 200 nm SUVs for both experiment and simulation. All sensitivities were compared at concentrations that gave an average membrane coverage of approximately 3%. The concentrations of protein in solution required to achieve this level of membrane coverage was 5 nM for the 10 and 150 mM NaCl conditions, and 10 nM for the 450 mM NaCl condition. Error bars are the 95% confidence interval of the mean. Each bin contains *N* = 36–249 data points acquired cumulatively from three independent replicates. **c** Simulated surface with representative IDP conformations. (Top) The orange segment represents the first 20 monomer units within the polymer. The configuration of this segment was chosen in previous steps of the simulation and is fixed in all subsequent steps. Three of the 10,000+ segment conformations generated for the remainder of the polymer chain are shown in green. (Bottom) When radius of curvature for the surface is increased, one of the three conformations becomes unallowable (red). **d** Schematic of proposed entropy-driven curving sensing mechanism for IDPs

To examine the relative contributions of the chain entropy and chain–chain repulsion mechanisms to curvature sensitivity, we measured the curvature sensitivity of membrane-bound AP180CTD as a function of salt concentration. First we used fluorescence correlation spectroscopy to measure the impact of sodium chloride concentration, ranging from 10 to 450 mM, on the relative dimensions of AP180CTD proteins diffusing freely in 3D solution (Supplementary Figs. 12 and 13). Figure 3a and Supplementary Table 1 show that the hydrodynamic radius of AP180CTD decreases from 6.7 to 5.6 nm with increasing salt concentration in this range. In contrast, transferrin, which we used as a globular control protein, changed negligibly in size over the same range of salt concentration (Supplementary Figures 13g-h and Supplementary Table 1). This result suggests that increasing salt concentration decreases the chain rigidity of AP180CTD. Next we compared the sensitivity of AP180CTD to membrane curvature over the same range of salt concentration. As displayed in Fig. 3b, relative curvature sensitivity increased approximately two-fold when the concentration of sodium chloride in the surrounding solution was raised from 10 to 450 mM. This result suggests that the impact of membrane curvature on chain entropy, rather than on chain–chain repulsion, is the dominant mechanism of membrane curvature sensing by AP180CTD. Nonetheless, electrostatic interactions play an important role because residue–residue repulsion within the IDP chain controls chain rigidity.

Next we used Monte Carlo simulations to evaluate whether increases in chain conformational entropy with increasing substrate curvature were sufficient to explain our experimental data. Specifically, we calculated the changes in entropy for polymer chains tethered to convex surfaces, where the polymer conformations were determined by a self-avoiding walk (SAW) model. Since coverage of the membrane surface by proteins is low in these experiments, it is reasonable to assume that membrane curvature does not change significantly upon protein binding, as justified in the Supplementary Discussion section. Based on this assumption, changes in membrane entropy upon protein binding were not incorporated into our simulations. This assumption is further justified by the observation that the mean vesicle diameter did not shift upon exposure to his-AP180CTD (Supplementary Fig. 17), as would have been expected had AP180CTD molecules become crowded on membrane surfaces, resulting in high steric pressure^26^. Nonetheless, we cannot rule out the possibility that protein-membrane interactions could locally modify the membrane entropy by influencing membrane mechanical properties.

Under the assumption that curvature is not altered by protein binding, the number of allowable chain configurations decreases with decreasing substrate curvature as depicted in Fig. 3c. Using Monte Carlo simulations, and deploying a diverse class of SAW models with varying rigidity, we calculated *R*_g_ for AP180CTD, for which the experimental *R*_g_ is known (Supplementary Figs. 18-22 and Supplementary Table 2). The optimal SAW model was chosen based on the best agreement between the calculated and measured *R*_g_ for AP180CTD at 150 mM NaCl concentration (Supplementary Figs. 12-13). This model provided an estimate of the Kuhn length (*l*_K_) at 150 mM NaCl, 0.43 nm, which we used to obtain entropy changes as a function of radius of curvature of the tethering substrate, employing the Hypothetical Scanning Monte Carlo (HSMC) method^27^. Assuming a length per amino acid of 0.38 nm^28,29^, and holding the IDP contour length constant at 216 nm, *l*_K_ was varied to capture the impact of salt concentration on AP180CTD. The results of these simulations indicate that the entropy for IDP chains bound to 40 nm diameter vesicles relative to chains bound to 200 nm diameter vesicles increases by approximately a factor of 2 as *l*_K_ increases from 0.36 to 0.66 nm (Supplementary Fig. 23). Using a simple Boltzmann formula to relate chain entropy and binding probability (Eq. 12), these results predict that relative curvature sensitivity over the same range of vesicle diameter should decrease by a similar factor with increasing *l*_K_. These values are in reasonable agreement with the experimentally measured curvature sensitivities, as shown in Fig. 3b. Specifically, *l*_k_ values of 0.66, 0.43, and 0.36 nm provide the best approximation of the measured curvature sensitivities under conditions of 10, 150, and 450 mM sodium chloride, respectively. Further, the average radius of the chain must increase as *l*_K_ increases, for fixed total length of the chain^30^. In agreement with this principle, the results of the Monte Carlo simulation for IDP chains fluctuating freely in solution provides reasonable agreement with the experimentally determined decrease in AP180CTD hydrodynamic radius with increasing salt concentration, as illustrated in Fig. 3a. This agreement further illustrates that screening of residue–residue electrostatic interactions tunes the rigidity and conformational entropy of the polymer-like IDP chain. This agreement is expected whenever IDPs behave as self-avoiding polymers in solution, as shown by comparing simulation results to polymer theory for a SAW^30^, Supplementary Figure 20. Collectively, the comparison of experiments and simulations shows that curvature sensing by the disordered domain of AP180 arises primarily from the influence of substrate curvature on the conformational entropy of the polymer-like IDP chain (Fig. 1d).

### Disordered domains sense the curvature of filopodia

**Fig. 4:**
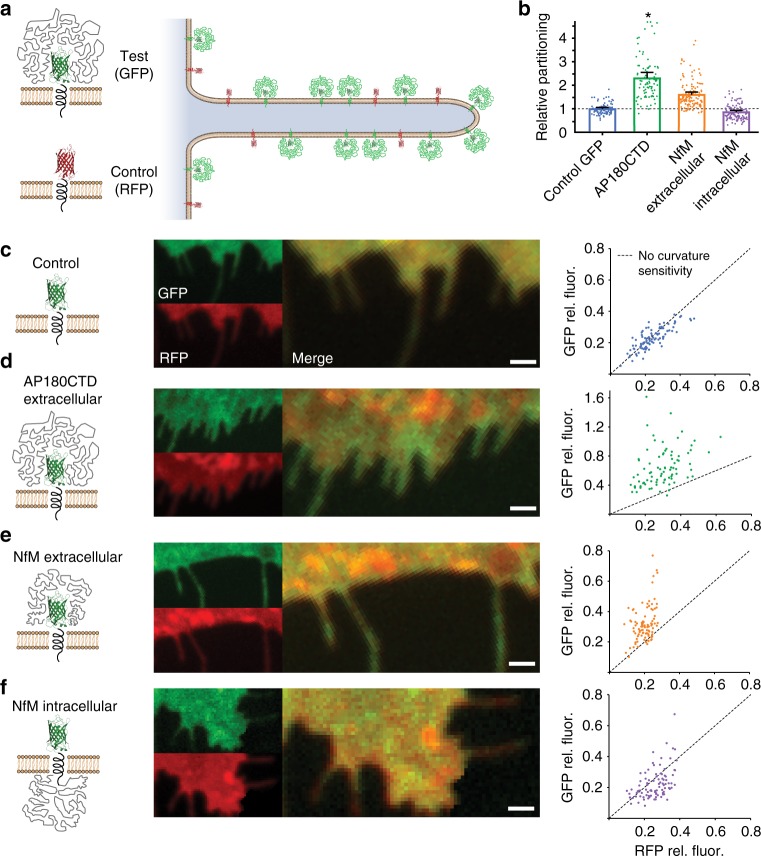
Disordered proteins sense the curvature of filopodia in live cells. **a** Schematic of GFP and RFP partitioning on the outer membrane surface of live cells in the presence of filopodia when an IDP is attached to GFP. **b** Average change in filopodia partitioning for GFP-labeled proteins relative to RFP-labeled proteins. Error bars represent the 95% confidence interval of the mean for all filopodia within a given condition. *N* = 94, 85, 132, and 111 filopodia measured for control GFP, AP180CTD, NfM extracellular, and NfM intracellular proteins, respectively. Asterisk denotes two data points for the AP180CTD data set that extended beyond the range of the plot, having values of 5.8 and 7.8. **c**–**f** Structure, fluorescence microscopy images of filopodia, and plots of relative GFP versus RFP fluorescence on filopodia for control GFP, extracellular AP180CTD, extracellular NfM, and intracellular NfM, respectively. Scale bars in (**c**–**f**) represent 1 µm

Having characterized the molecular mechanism of curvature sensing by disordered domains in vitro, we next asked whether disordered domains are capable of sensing membrane curvature in the crowded, heterogeneous environment of the cellular plasma membrane. Here we utilized a recently developed assay^31^ to quantify the partitioning of transmembrane fusion proteins between filopodia, which have an average diameter of approximately 100–200 nm,^32^ and the comparatively flat plasma membrane surface (Fig. 4a). The results of this analysis are summarized in Fig. 4b. We first examined the relative partitioning of two control proteins, both consisting of the transmembrane domain of the transferrin receptor fused at its C-terminus to a fluorescent protein domain, GFP or RFP, such that fluorescent protein domain was displayed on the outer plasma membrane surface, Fig. 4a, c. We expressed both control proteins simultaneously in retinal pigmented epithelia (RPE) cells and imaged filopodial structures at the coverslip surface using two channel confocal fluorescence microscopy. Since these proteins have nearly identical morphologies, we expected them to partition very similarly between filopodia and the plasma membrane. Indeed, when the GFP and RFP images were overlaid, both the plasma membrane and filopodia appeared yellow, indicating a similar ratio of filopodia to plasma membrane partitioning for both control proteins (Fig. 4c, center). To quantify the partitioning across multiple cells and filopodia, we plotted the filopodial intensity relative to the local plasma membrane intensity for the GFP control protein versus that of the RFP control protein (Fig. 4c, right). As expected, these data fall along a line with slope equal to one, indicating that the two proteins partition equivalently to the filopodia (Fig. 4b).

Having established these control data, we next fused the disordered domain of AP180 (AP180CTD) to the c-terminus of the GFP control protein, resulting in the display of AP180CTD on the outer plasma membrane surface (Fig. 4d). In cells that simultaneously expressed this protein and the RFP control protein, filopodia appeared green in overlaid images, suggesting that extracellular display of AP180 increased partitioning to filopodia (Fig. 4d, center). Similarly, nearly all filopodia had GFP to RFP relative intensity ratios above one (Fig. 4d, right), with an average value of 2.34 + 0.12 (Fig. 4b). This finding is in reasonable agreement with theoretically expected partitioning for membrane surfaces of 100–200 nm diameter, approximately two fold (Supplementary Table 3).

If disordered domains drive increased partitioning to the convex outer surfaces of filopodia, then displaying the same domain on the concave inner filopodial surface should drive a decrease in partitioning. To test this idea, we modified the GFP control protein to display the disordered domain of Neurofilament-M (NfM) on either the outer (C-terminal fusion) or inner (N-terminal fusion) plasma membrane surface (Fig. 4e, f, respectively). Here NfM was chosen because it is a well-characterized intrinsically disordered domain that is known to be highly water soluble^33^ and because it has no known cytoplasmic binding partners in non-neuronal cells. Notably, the many endocytic binding partners of AP180CTD^34^ would likely alter its physical properties and therefore preclude its use in this experiment. As expected display of NfM on the outer plasma membrane surface drove a partitioning factor greater than one, 1.59 + 0.05, while display of the same domain on the inner surface resulted in a value less than one, 0.85 + 0.03 (Fig. 4b, e, f). Notably, the smaller partitioning factor of extracellular NfM relative to extracellular AP180CTD is expected owing to the shorter length of NfM, 438 amino acids versus 569 amino acids, which should proportionally reduce its conformational entropy^13^. Collectively, these results demonstrate that disordered protein domains can sense membrane curvature within the complex environment of a live cell.

### Disordered domains enhance sensitivity of structured domains

**Fig. 5:**
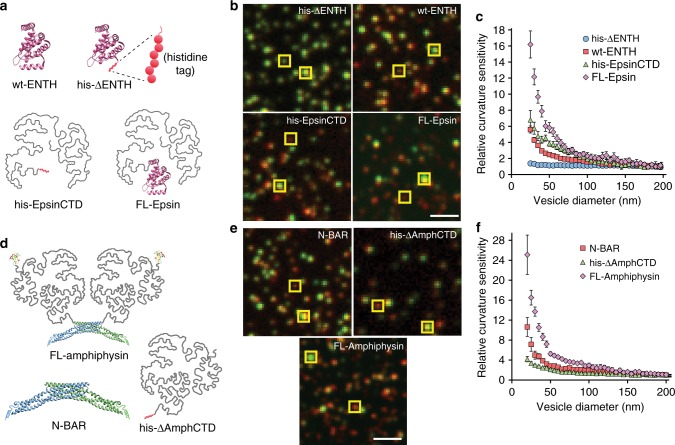
Disordered domains enhance curvature sensitivity of structured domains. **a** Schematic of Epsin1 domains and full-length protein. **b** Lipid, protein, and merged confocal fluorescent images of SUVs (30 nm extruded) and bound proteins. Yellow boxes highlight SUVs of varying brightness, corresponding to varying diameter. Protein (red) to lipid (green) fluorescence ratio appears higher for smaller (less bright in lipid channel) SUVs. SUVs used for his-ΔENTH and his-EpsinCTD binding contained 80% DOPC, 16% DGS-NTA, 2% DP-EG10-biotin, and 2% Oregon Green-DHPE. SUVs used for wt-ENTH and FL-Epsin binding contained 88.5% DOPC, 7.5% PI(4,5)P2, 2% DP-EG10-biotin, and 2% Oregon Green-DHPE. All proteins (**b**, **e**) were labeled with ATTO-594 dye. **c** Relative curvature sensitivities for the Epsin1 domains and full-length Epsin1 over a 25–200 nm SUV diameter range. All proteins were compared at concentrations that gave an average membrane coverage of approximately 3% (5 nM for his-ΔENTH, 450 nM for wt-ENTH, 500 nM for his-EpsinCTD, and 300 nM for FL-Epsin). **d** Schematic of Amphiphysin1 domains and full-length protein. **e** Lipid, protein, and merged fluorescent channels of SUVs (30 nm extruded) and bound proteins. Yellow boxes highlight SUVs of varying brightness as in (**b**). SUVs used for his-ΔAmphCTD binding contained 80% DOPC, 16% DGS-NTA, 2% DP-EG10-biotin, and 2% Oregon Green-DHPE. SUVs used for N-BAR and FL-Amphiphysin binding contained 76% DOPC, 15% DOPS, 5% PI(4,5)P2, 2% DP-EG10-biotin, and 2% Oregon Green-DHPE. **f** Relative curvature sensitivities for the Amphiphysin1 domains and full-length Amphiphysin1 over a 20–200 nm SUV diameter range. All proteins were compared at concentrations that gave an average membrane coverage of approximately 5% on 200 nm SUVs (10 nM for N-BAR, 10 nM for his-ΔAmphCTD, 25 nM for FL-Amphiphysin). Error bars in (**c**, **f**) are the 95% confidence interval of the mean. Each bin contains *N* = 33–358 data points acquired cumulatively from three independent replicates. Scale bars represents 2 μm

Having demonstrated that isolated IDPs domains are capable of sensing membrane curvature, we next asked whether disordered domains contribute to the curvature sensing abilities of other endocytic proteins. Specifically, we examined the endocytic adaptor proteins Epsin1 and Amphiphysin1, both of which consist of N-terminal structure-based curvature sensors followed by bulky, intrinsically disordered C-terminal domains (Fig. 5). To examine the ability of Epsin1’s C-terminal domain to sense membrane curvature, we expressed and purified his-EpsinCTD, which consisted of an N-terminal histidine tag followed by residues 144–575 of Epsin1 (*Rattus norvegicus*). We also expressed and purified the full-length Epsin1 protein, FL-Epsin (Fig. 5a). We estimated the projected area of Epsin1’s IDP domain on the membrane surface at approximately 85 nm^2^, based on our measurement of the projected area for his-AP180CTD (115 nm^2^), assuming that projected area scales approximately linearly with amino acid chain length, as would be the case for a random polymer.

We used the tethered vesicle assay to measure the dependence of protein binding on vesicle diameter (Fig. 5b). His-ΔENTH exhibited no apparent curvature sensitivity as described in Fig. 1e. In contrast, wt-ENTH, his-EpsinCTD, and FL-Epsin all appeared to sense membrane curvature as indicated by the increase in lipid channel (green) to protein channel (red) fluorescence intensity ratio with increasing vesicle diameter (Fig. 5b). To quantify the level of curvature sensitivity for each protein, we plotted normalized curvature sensitivity as a function of SUV diameter, under conditions for which all proteins had an average membrane coverage of approximately 3% (Fig. 5c). The wt-ENTH protein bound six-fold more densely to vesicles of 25 nm diameter in comparison to vesicles of 200 nm diameter. In contrast, his-EpsinCTD achieved a seven-fold increase in binding over the same range of SUV diameter. Interestingly, curvature sensitivity for FL-Epsin achieved a 16-fold increase in binding, clearly demonstrating that the presence of both the ENTH and disordered domains amplifies membrane curvature sensing.

We next examined Amphiphysin1 (*Homo sapiens)*, which consists of an N-terminal crescent-shaped BAR domain (N-BAR) of approximately 240 amino acids, followed by an intrinsically disordered domain of approximately 380 amino acids, followed by a small, structured SH3 domain of approximately 72 amino acids. The N-BAR domain has been previously demonstrated to sense membrane curvature, likely through a combination of membrane scaffolding by the curved BAR domain and insertion of the N-terminal amphipathic helix^4,35^. However, the contribution of the disordered domain to curvature sensing by Amphiphysin1 has not been previously examined. We quantified curvature sensing by each of Amphiphysin1’s component domains, including N-BAR (residues 1–242), the disordered domain, His-ΔAmphCTD, (residues 240–622), and the full-length protein, FL-Amphiphysin, (residues 1–695) (Fig. 5d). To isolate the contribution of the disordered domain, the SH3 domain of Amphiphysin1 (residues 623–695) was not included in the his-ΔAmphCTD protein.

We estimated the projected area of Amphiphysin1’s IDP domain as 75 nm^2^, scaling from the projected area for his-AP180CTD. In contrast, the N-BAR domain has a projected area of approximately 24 nm^2^ per monomer, based on its crystal structure and as assumed elsewhere^36^. N-BAR, his-ΔAmphCTD, and FL-Amphiphysin all appeared to sense membrane curvature as indicated by the increase in lipid channel (green) to protein channel (red) fluorescence intensity ratio with increasing vesicle diameter (Fig. 5e). To quantify the level of curvature sensitivity for each protein, we plotted normalized sensitivity as a function of SUV diameter under conditions for which all proteins had an average membrane coverage of approximately 5% (Fig. 5f). The N-BAR protein bound 11-fold more densely to vesicles of 20 nm diameter in comparison to vesicles of 200 nm diameter. By comparison his-ΔAmphCTD achieved a five-fold increase in binding over the same range of vesicle diameter. Strikingly, FL-Amphiphysin exhibited a substantially larger degree of curvature sensitivity in comparison to its constitutive domains, increasing 25-fold in bound density. This non-additive increase in curvature sensitivity upon inclusion of the disordered domains suggests a synergistic interaction among structured domains and disordered domains. One possible explanation for this effect is the close proximity of the disordered domains in the Amphiphysin1 dimer, which are approximately 6 nm apart, based on the protein structure^4^, similar to the hydrodynamic radii of the disordered domains. As a result of this close spacing, we would expect the disordered domains to sterically and electrostatically exclude one another when the dimer binds to the membrane surface. These IDP–IDP interactions likely contribute to the substantial increase in membrane curvature sensitivity for the full-length Amphiphysin1 protein in comparison to either the N-BAR or the disordered domain alone. Although the membrane binding mechanisms for wt-ENTH domains, N-BAR domains, and hexa-histidine tags are each different, we controlled for their varying membrane affinities by adjusting their concentration in solution such that each protein reached the same average coverage of the membrane surface. Nonetheless, we cannot rule out the possibility that the membrane chemical potential may vary to some degree among the different mechanisms of protein binding.

## Discussion

Here we have demonstrated that the IDP domains of AP180, Neurofilament-M, Epsin1, and Amphipysin1 sense membrane curvature at comparable levels to structured curvature sensing domains and are capable of synergistically amplifying the sensitivity of these domains. Further, by comparing our experimental results to Monte Carlo simulations, we determined that the curvature sensitivity of disordered domains arises primarily from the impact of substrate curvature on their conformational entropy. In contrast to previously identified curvature sensors that rely on specific structural motifs, our findings illustrate that the lack of a well-defined three dimensional structure can itself provide a potent mechanism for sensing membrane curvature.

Notably, the mechanism of curvature sensing identified here arises from the entropy of the amino acid chain and is therefore unique to disordered proteins. This mechanism is fundamentally distinct from previously identified mechanisms of membrane bending by both structured and disordered domains, which arise from molecular crowding at membrane surfaces^21,37^. In particular, curvature generation by molecular crowding of IDPs is expected only when proteins cover a large fraction of the membrane surface^21^. In contrast, the curvature sensing mechanism reported here is only prominent when membrane coverage is low and becomes less potent as membrane coverages increases (Fig. 1f).

This entropic mechanism identified here could apply to any of the substantial group of IDP domains that display random coil-like behavior^38^. Specifically, high proline content^39^ and high net charge^40^ are common features among endocytic proteins^16^, which have frequently been associated with intrinsic disorder and extended peptide chain conformations. In particular, the FBAR-containing proteins FCHo1/2, which initiate coated-vesicle assembly^41^, and FBP17^42^ and CIP4^43^, which participate in vesicle departure, have a domain morphology similar to amphiphysin, where large IDP domains are locally clustered by the structured BAR domain scaffold. Furthermore, Hsc70, the uncoating ATPase which has recently been shown to apply entropic pressure during clathrin coat disassembly^44^, is recruited to clathrin-coated vesicles by auxilin^45^, which also has substantial IDP content. Further, protein constituents of the Coat Protein I and II pathways also contain significant IDP content^11^, suggesting a possible role in membrane curvature sensing. Interestingly, IBAR domains such as IRSp53, which have a concave structure and concentrate in filopodia^46^, do not contain significant disordered domains. Such domains would presumably work against the ability of IBAR to sense concave membrane curvature. Finally, disordered domains are ubiquitous among transmembrane proteins^47^, such as G protein-coupled receptors^48^ and the components of highly curved synaptic vesicles^49^, suggesting that disordered domains may help transmembrane proteins sense membrane curvature, in addition to recent reports of curvature sensing by specific transmembrane protein structural features^31,50,51^.

Entropically driven curvature sensing by disordered domains fundamentally departs from the deterministic structure-function paradigm that presently dominates our understanding of membrane curvature sensing. Specifically, the findings of this work suggest that disordered domains play important roles by collaborating with structured domains to detect curved membrane sites during diverse biological processes.

## Methods

### Materials

1,2-dioleoyl-sn-glycero-3-phosphocholine (DOPC), 1,2-dioleoyl-sn-glycero-3-phospho-L-serine (DOPS), L-α-phosphatidylinositol-4,5-biphosphate (PI(4,5)P2), and 1,2-dioleoyl-sn-glycero-3-[(N-(5-amino-1-carboxypentyl)iminodiacetic acid)succinyl] (DGS NTA), were purchased from Avanti Polar Lipids, Inc. Dipalmitoyl-decaethylene glycol-biotin (DP-EG10-biotin) was generously provided by D. Sasaki from Sandia National Laboratories, Livermore, CA^52^. Oregon Green® 488 1,2,-dihexadecanoyl-sn-glycero-3-phosphoethanolamine (OG-DHPE) and neutravidin were purchased from Thermo Fisher Scientific. Ethylenediaminetetraacetic acid (EDTA), ethylene glycol tetraacetic acid (EGTA), Tris(2-carboxyethyl) phosphine hydrochloride (TCEP), phenylmethanesulfonyl fluoride (PMSF), EDTA-free protease inhibitor tablets, imidazole, poly-L-lysine (PLL), ATTO-488 NHS-ester, and ATTO-594 NHS-ester were purchased from Sigma-Aldrich. Sodium chloride, 4-(2-hydroxyethyl)-1-piparazineethanesulphonic acid (HEPES), isopropyl-β-D-thiogalactopyranoside (IPTG), β-mercaptoethanol, and Triton X-100 were purchased from Fisher Scientific. Amine reactive PEG (mPEG-Succinimidyl Valerate MW 5000) and PEG-biotin (Biotin-PEG SVA, MW 5000) were purchased from Laysan Bio, Inc. All reagents were used without additional purification.

### Plasmids for tethered vesicle assay

DNA plasmids for his-AP180CTD (rat AP180, amino acids 328-896; CAA48748), his-EpsinCTD (rat Epsin1, amino acids 144-575; EDL75887), and his-Epsin-FL (rat Epsin1, amino acids 1-575; EDL75887) in pET32c vectors were kindly provided by E. Ungewickell, Hannover Medical School, Germany. Constructs coding for his-AP180CTD and his-EpsinCTD in pGex4T2 vectors were previously described^21^. A construct coding for Epsin-FL was obtained by creating a plasmid coding for GST-Epsin-FL. PCR amplified regions of the pET32c vector coding for his-Epsin-FL were cloned into a pGex4T2 vector using incorporated restriction sites EcoRI and XhoI (primers in Supplementary Table 4). The vector containing C-terminally located histidine tag for AP180CTD (GST-AP180CTD-his) was formed by incorporating a 6-his tag coding sequence in the reverse primer used to amplify AP180CTD. The product was then subcloned into the pGex4T2 vector using BamHI and EcoRI restriction sites (primers in Supplementary Table 4). The pGex4T2 plasmid containing wt-ENTH (rat Epsin1, amino acids 1-164; EDL75887) was a gift from H. McMahon, MRC Laboratory of Molecular Biology, Cambridge, UK^7^. We generated his-ΔENTH (rat Epsin1, amino acids 16-164; EDL75887) previously^37^. The pGex6P1 plasmid containing Amphiphysin-FL (human Amphiphysin1, amino acids 1-695; AAA21865) was a gift from T. Baumgart, University of Pennsylvania. The construct for the N-BAR domain (human Amphiphysin1, amino acids 2-242; AAA21865) was obtained by cloning the corresponding sequence of the Amphiphysin-FL plasmid into a pGex4T2 vector using incorporated restriction sites BamHI and EcoRI (primers in Supplementary Table 4). For the his-ΔAmphCTD construct (human Amphiphysin1, amino acids 240-622; AAA21865), the pGex4T2 vector containing his-AP180CTD was utilized. The sequence corresponding to AP180CTD was removed and the sequence coding for residues 240-622 of Amphiphysin was ligated into its place using the restriction sites SalI and XhoI (primers in Supplementary Table 4). This resulted in a pGex4T2 construct coding for hexa-histidine tagged AmphCTD, without its SH3 domain (amino acids 623-695).

### Plasmids for live cell assay

Plasmids for control GFP (TfR-Δecto-GFP), AP180CTD extracellular (TfR-Δecto-GFP-AP180CTD), and Neurofilament-M-CTD extracellular (TfR-Δecto-GFP-NfMCTD) fusion proteins were prepared as previously described^21^. All of these chimeric proteins contained the intracellular and transmembrane domains of transferrin receptor (TfR-Δecto, human, amino acids 1-88; AAA61153) and GFP (EGFP; AAB02574). For this present work, a A206K mutation was added to GFP to prevent dimerization of GFP-containing proteins^53^. The mutation was performed using site-directed mutagenesis (primers in Supplementary Table 4).

The control RFP construct was synthesized by modifying the TfR-Δecto-GFP described above, where GFP was replaced where RFP (Accession number ABN59525). AgeI and NotI restriction enzymes were used to excise GFP from the pEGFP-N1 vector containing TfR-Δecto-GFP (primers in Supplementary Table 4). Next, RFP was PCR amplified from the pcDNA3-mRFP plasmid acquired through Addgene as a gift from Dr. Douglas Golenbock (Addgene #13032). Finally, RFP was ligated into the TfR-Δecto backbone described above, yielding TfR-Δecto-RFP.

The NfM intracellular construct was also synthesized by modifying the TfR-Δecto-GFP described above. The EcoRI restriction enzyme was used to digest the pEGFP-N1 vector containing TfR-Δecto-GFP. Next, the CTD of NfM was PCR amplified from the pEGFP-mNFM vector (Addgene #32909) using primers that incorporated the EcoRI restriction site (primers in Supplementary Table 4). The PCR product was then digested with the EcoRI restriction enzyme and ligated into the TfR-Δecto-GFP backbone.

### Protein expression and purification

Epsin-FL, wt-ENTH, his-EpsinCTD, Amphiphysin-FL, his-ΔAmphCTD, and N-BAR were expressed as fusion proteins containing N-terminal glutathione-S-transferase (GST) in *E. coli* BL21 (DE3) pLysS cells (Invitrogen). For wt-ENTH, induction was carried out with 100 µM IPTG at 37 °C for 3 h. For the other constructs, induction was carried out with 1 mM IPTG at 30 °C for 6 h. Bacteria were then lysed with buffer consisting of 500 mM Tris, 5 mM EDTA, 10 mM β-mercaptoethanol, 1 mM PMSF, 5%v/v glycerol, 1%v/v Triton X-100, and 1x Roche protease inhibitor cocktail (Sigma-Aldrich) (pH 8.0). Next, bacteria were sonicated on ice (3 × 2000 joules). Bacterial lysate was then separated using ultracentrifugation at 103,800 × *g* for 40 min. GST-containing fusion proteins were then purified by incubation with glutathione agarose beads (Thermo Fisher Scientific). Bound proteins were thoroughly washed with buffer consisting of 25 mM HEPES, 150 mM NaCl, 10 mM β-mercaptoethanol, and 1 mM EDTA (pH 7.4). All proteins, with the exception of Amphiphysin-FL, were then cleaved from the beads with thrombin (GE Healthcare Life Sciences) overnight at 15 °C. Thrombin was subsequently removed using *p*-aminobenzamidine-agarose (Sigma-Aldrich) for 30 min at 4 °C. Amphiphysin-FL was cleaved from agarose beads using the protease HRV-3C (Thermo Scientific Pierce). The GST-containing HRV-3C was removed using glutathione resin.

The C-terminal domain of AP180 (his-AP180CTD) was expressed as a GST fusion protein for increased stability during expression, and the GST was subsequently removed by thrombin cleavage. BL21 transformed with the expression construct were grown in 2X YT media at 30 °C, induced with 1 mM IPTG at an OD600 of 0.8, and grown for an additional 7 h at 30 °C. Cell pellets from a 2 L culture were resuspended in 100 ml of 0.5 M Tris pH 8.0, 5 mM EDTA, 5% glycerol, 5 mM TCEP, and 2 Roche protease inhibitor cocktail pellets (Roche #5056489001) (lysis buffer) by sonication on ice (4 × 2000 joules), followed by the addition of Triton X-100 to 1%. All subsequent steps were carried out at 4 °C. The lysate was clarified by ultracentrifugation at 186,010 × *g* for 30 min and the supernatant was applied to 15 ml of glutathione Sepharose (GE Healthcare #17-0756-05), washed with 10 column volumes of lysis buffer, and eluted with 15 mM glutathione in lysis buffer. The buffer was exchanged with 50 mM Tris pH 8.0, 10 mM CaCl_2_ (thrombin digestion buffer) in a Zeba Desalting Column (Thermo Scientific #89891), and the GST tag was cleaved with the Thrombin CleanCleave kit (Sigma-Aldrich #RECOMT-1KT) for 14 h at 4 °C in 50 mM Tris, pH 8.0 and 10 mM CaCl_2_ with rocking, followed by the addition of 15 mM EGTA, 5 mM EDTA, 150 mM NaCl, and 5 mM TCEP. The GST was removed by running the digestion products through a glutathione Sepharose column. The purified protein was concentrated to 3.6 mg ml^−1^ in an Amicon Ultra-15 centrifugal filter (MilliporeSigma #UFC903024), and stored as liquid nitrogen pellets at −80 °C.

Hexa-histidine tagged ENTH (his-ΔENTH) was expressed in BL21 cells following overnight induction with 1 mM IPTG at 18 °C. Bacterial lysate was incubated in Ni-NTA agarose beads (Qiagen) with buffer consisting of 25 mM HEPES, 150 mM NaCl, and 5 mM β-mercaptoethanol (pH 7.4). After thorough washing, his-ΔENTH was eluted from the resin by incorporating imidazole into the buffer. The imidazole concentration was gradually increased in a step-wise manner up to a final concentration of 200 mM. The eluted protein was then concentrated and dialyzed against the original incubation buffer at 4 °C overnight. After purification, all proteins were stored at −80 °C.

### Protein labeling

ATTO-NHS dyes were dissolved in DMSO at a concentration of 10 mM and stored at −80 °C. Labelling of lysine residues was performed with protein concentrations ranging from 20 to 100 μM in buffer consisting of 25 mM HEPES, 150 mM NaCl, and 5 mM TCEP (pH 7.4). Dye was added to the protein in 2–3× stoichiometric excess and allowed to react for 1 h on ice, resulting in labelling ratios ranging from 0.5:1 to 1.5:1 dye:protein. The amount of DMSO in the labelling reaction mixture never exceeded 1%v/v. Unconjugated dye was separated from labeled protein using size exclusion chromatography with Sephadex G-25 (GE Healthcare Life Sciences) for wt-ENTH, his-ΔENTH, and BAR. For all other proteins, Centri-Spin size exclusion columns (Princeton Separations) were used. Protein and dye concentrations were monitored using UV–Vis spectroscopy. Labelled proteins were stored at −80 °C.

### GFP purification and PEG conjugation

GFP and PEG40K-GFP were prepared as previously described^53^. Briefly, non-dimerizable hexa-his-tagged EGFP A206K (his-GFP; AAB02574) was made suitable for maleimide-PEG conjugation by mutating an exposed glycine residue in the N-terminal linker region to a cysteine. After expression, bacterial lysates were incubated with Ni-NTA agarose in 20 mM HEPES, 150 mM NaCl (pH = 7.4) buffer, thoroughly washed, and eluted using 200 mM imidazole buffer. His-GFP was then dialyzed to remove imidazole. For PEG conjugation, his-GFP was incubated with an excess of maleimide-PEG40K at 4 °C overnight in 20 mM phosphate, 150 mM NaCl, 1 mM TCEP (pH = 7.4) buffer. The reaction mixture was then incubated in Ni-NTA resin, washed, and eluted to remove unreacted PEG. The eluted protein was then subjected to size exclusion chromatography using a Sepharose G75 column to remove unreacted protein.

### Preparation of small unilamellar vesicles

Lipid aliquots (stored at −80 °C) were brought to room temperature and combined in appropriate quantities (see below*) to achieve the desired stoichiometric ratios. Once combined, solvent was evaporated using a gentle nitrogen stream. The resulting lipid film was further dried under vacuum overnight. The lipid film was then hydrated in appropriate buffer (see below**) at a final concentration of 200 μM total lipid, thoroughly mixed, and held at room temperature for 15 min while lipids were allowed to hydrate. Small unilamellar vesicles (SUVs) were made using three different methods: probe tip sonication (Branson Ultrasonics), extrusion through a 30 nm polycarbonate membrane (Whatman plc), and extrusion through a 200 nm polycarbonate membrane (Whatman plc). The three preparations resulted in SUV populations with average diameters of 49 + 5 nm, 67 + 4 nm, and 147 + 7 nm, respectively, as determined by dynamic light scattering.

*The molar composition of SUVs used for histidine tagged proteins was as follows: 80% DOPC, 16% DGS NTA, 2% DP-EG10-biotin, 2% OG-DHPE. For SUVs used in wt-ENTH and Epsin-FL binding, the molar composition was 88.5% DOPC, 7.5% PI(4,5)P2, 2% DP-EG10-biotin, 2% OG-DHPE. For N-BAR and Amphiphysin-FL binding, the SUV molar composition used was 76% DOPC, 15% DOPS, 5% PI(4,5)P2, 2% DP-EG10-biotin, 2% OG-DHPE.

**For histidine tagged proteins, SUVs were made in a buffer consisting of 25 mM HEPES and 150 mM NaCl (pH 7.4). For all other proteins, SUVs were made in a buffer consisting of 25 mM HEPES, 150 mM NaCl, 0.5 mM EDTA, and 0.5 mM EGTA (pH 7.4).

### Tethering of lipid vesicles and protein binding

SUVs were tethered as previously described with slight modification^26^. Briefly, glass cover slips were passivated with a layer of biotinylated PLL-PEG, 5 kDa PEG. SUVs doped with 2 mol% DP-EG10-biotin were then tethered to the passivated surface using NeutrAvidin.

PLL-PEG was synthesized by combining amine reactive PEG and PEG-biotin in molar ratios of 98% and 2%, respectively. This PEG mixture was added to a 20 mg/mL mixture of PLL in 50 mM sodium tetraborate (pH 8.5) such that the molar ratio of lysine subunits to PEG was 5:1. The mixture was continuously stirred at room temperature for 6 h then buffer exchanged into 25 mM HEPES, 150 mM NaCl (pH 7.4) using Centri-Spin size exclusion columns (Princeton Separations). Imaging wells consisted 5 mm diameter holes in 0.8 mm thick silicone gaskets (Grace Bio-Labs). Gaskets were placed directly on top of no. 1.5 glass cover slips (VWR International), creating a temporary water-proof seal. Prior to well assembly, gaskets and cover slips were cleaned in 2%v/v Hellmanex III (Hellma Analytics) solution, rinsed thoroughly with water, and dried under a nitrogen stream. To each dry imaging well, 20 µL of PLL-PEG was added. After 20 min of incubation, wells were serially rinsed with 25 mM HEPES, 150 mM NaCl (pH 7.4) using gentle pipetting until a 15,000-fold dilution was achieved. Next, 4 µg of NeutrAvidin dissolved in 25 mM HEPES, 150 mM NaCl (pH 7.4) was added to each sample well and allowed to incubate for 10 min. Wells were then rinsed with the appropriate buffer to remove excess NeutrAvidin. Sonicated, 30 nm-extruded, and 200 nm-extruded SUVs were then each independently added (see below*) to sample wells at lipid concentrations of 0.5, 1, and 5 µM, respectively, and allowed to incubate for 10 min. Excess SUVs were then rinsed from the well using the appropriate buffer. Finally, specific proteins at desired concentrations in appropriate buffers—with the addition of 5 mM TCEP— were added to wells and allowed to incubate for at least 30 min before imaging. All proteins were labeled with ATTO-594. During all rinsing and reagent addition steps, proper precaution was taken to ensure that changes in sample volume within the imaging wells were minimized.

*For each protein at any given concentration, SUV binding was observed independently on sonicated, 30 nm-extruded, and 200 nm-extruded SUV populations in three separate wells. The resulting data from the three experiments was then consolidated to cover a broad range of vesicle diameter.

### Cell culture and transfection

Human RPE cells (ARPE-19) were purchased from American Type Culture Collection. Cells were grown in 1:1 F12:DMEM supplemented with 10% FBS, 20 mM HEPES, Pen/Strep/L-glutamine (100 units/ml, 100 μg ml^−^^1^, 300 μg ml^−^^1^ respectively) and incubated at 37 °C with 5% CO_2_. Cells were seeded onto acid washed coverslips at a density of 5 × 10^4^ cells per coverslip for 24 h before transfection with 1 µg of plasmid DNA in 3 µL of Fugene transfection reagent (Promega, Madison, WI, USA). Cells were imaged 20 h after transfection.

### Fluorescence microscopy

A spinning disc confocal microscope (Zeiss Axio Observer Z1 with Yokagawa CSU-X1M) was used to visualize tethered SUVs and bound proteins. For excitation, lasers with wavelengths of 488 and 561 nm were used. Emission filters were centered at 525 nm with 50 nm width and 629 nm with a 62 nm width. A triple pass dichroic mirror was used: 405/488/561 nm. A Plan-Apochromat 100×, 1.4 numerical aperture oil immersion objective was used. Tethered SUVs and bound proteins were imaged on a cooled (−70 °C) EMCCD iXon3 897 camera (Andor Technology; Belfast, UK).

### Image processing for tethered vesicles

Image stacks perpendicular to the sample plane (16 slices, 0.1 µm steps) of lipid and protein fluorescence were acquired using confocal microscopy. Images were 513 × 513 pixels (1 pixel = 133.3 nm) in size. In all images, the stacks were cropped to the center 171 × 171 pixels (center 1/9th) to ensure uniform focus and the frame with the largest mean brightness was selected as the best focus image for analysis. Since all of the SUVs of interest (20–250 nM diameter) appeared as diffraction-limited puncta, publically-available particle detection software (cmeAnalysis) was used to obtain fluorescence amplitudes^54^. Individual vesicles were detected by fitting two-dimensional Gaussian profiles to each puncta in the lipid fluorescence channel. The standard deviation of the Gaussian was determined from the point spread function of our microscope. If a detected punctum had an amplitude significantly above its local fluorescence background, it was included in the analysis. The program then used an algorithm to find puncta in the fluorescent protein channel that were spatially colocalized with the centroids of the puncta in the fluorescent lipid channel. The dimension of this search region was three times the standard deviation of the Gaussian fit to the point spread function. In the event that a protein punctum could not be found in a search region, protein fluorescence in that region was fit with a two-dimensional Gaussian with a centroid corresponding that of the lipid punctum. This step in the analysis takes into account SUVs that did not have any bound protein. To further ensure that all reported lipid puncta included in the analysis were above the noise threshold of our images, we only accepted puncta in the lipid channel that persisted in the same location throughout three consecutive frames in the image stack.

### Calibration of vesicle size and protein number

SUV diameter was estimated on the basis of fluorescence in the lipid channel confocal images, using a previously reported protocol^5^. Briefly, we computed a scaling factor which centered the peak of the vesicle brightness distribution, prior to protein addition, to the average vesicle diameter obtained from dynamic light scattering. The addition of proteins did not affect the vesicle brightness distribution over the concentration ranges observed. The number of bound proteins to each vesicle was estimated using single-molecule imaging to determine the brightness of a single ATTO-594 labelled protein. 50 pM of labelled protein was added to an imaging well consisting of freshly clean bare glass. After acquiring an image z-stack, the image with the brightest average pixel value was used as the best focus frame for analysis. We used cmeAnalysis to determine the brightness distribution of adsorbed single proteins which appeared as diffraction-limited puncta on the glass cover slip surface (Supplementary Fig. 9a-b). To ensure the presence of single molecules, the brightness distribution of puncta that underwent single-step photobleaching was also measured (Supplementary Fig. 9c). This distribution yielded a similar peak value, indicating agreement with the overall puncta brightness distribution. This peak value was then used to estimate the number of proteins bound to a given vesicle. Calibration values for ATT0594-labeled his-ENTH and his-AP180CTD were very similar, indicating that the protein single-molecule intensity was not a function of protein type. Typical experiments measuring SUV diameter and protein content generated in excess of 1000 data points per condition. To display these relationships, a moving average was applied to each data set as demonstrated in Supplementary Figures 5-8. Data was binned according to SUV diameter in increments of 5 + 2.5 nm for diameters ranging from 20 to 250 nm. Within each bin, the mean number of membrane-bound proteins was reported along with its standard error (95% confidence). This binned dataset was then used to calculate protein coverage and normalized coverage values. Coverage of 200 nm diameter SUVs was calculated as the average coverage from 190 to 210 nm. All other coverage values were normalized to this value, yielding the normalized coverage.

### Fluorescence correlation spectroscopy

Imaging wells were coated with supported lipid bilayers consisting of DOPC (1,2-dioleoyl-sn-glycero-3-phosphocholine) to block adsorption of protein from solution onto the coverslip surface. SUVs consisting of 100% DOPC were prepared by sonication in 25 mM HEPES, 150 mM NaCl (pH 7.4) and added to the imaging wells at a lipid concentration of 500 µM. After 10 min of incubation at room temperature, excess vesicles were thoroughly rinsed from the imaging well. Protein labelled with ATTO-488 dye was then added to the well at concentrations ranging from 0.5 to 2.0 nM. FCS measurements in solution, several micrometers above the lipid passivation layer were acquired using a custom-built time-correlated single-photon counting confocal microscope that has been described previously^21^. FCS autocorrelation curves were collected for 200 s using Becker and Hickl data acquisition software.

Six FCS curves were obtained for all AP180CTD containing samples, while three curves were obtained for all other proteins. Each curve was fit with the standard 2D autocorrelation function:

3 $$G\left( t \right){\mathrm{ = 1 + }}\left( {1 + Ae^{ - {\mathrm{t}}/\tau _c}} \right)\left( {\frac{1}{{N_p}}} \right)\left( {\frac{1}{{{\mathrm{1 + }}\left( {\frac{t}{{\tau _D}}} \right)^\alpha }}} \right)$$

where *N*_p_ corresponds to the average number of particles diffusing through the confocal volume and *τ*_D_ is the particle diffusion time. To improve the overall quality of our fits, we included an anomalous diffusion coefficient (*α*) and triple-state contribution terms *A* and *τ*_c_. Values for α varied between 0.9 and 1.0, minimally impacting the regressed values of *τ*_D_. Triplet-state variables *A* and *τ*_c_ were held constant at 0.05 and 5 μs^55^, respectively, and had very little effect on fitting the longer time diffusion processes of interest.

### Image analysis for live cell experiment

Image stacks taken at fixed distances perpendicular to the membrane plane (18 slices, 0.1 µm steps) were acquired using confocal microscopy. Images were 513 × 513 pixels (1 pixel = 133.3 nm) in size and the frame with the largest mean brightness was selected as the best focus image for analysis. Using ImageJ analysis software, an average fluorescence intensity on the area in the bulk cell membrane was determined for both GFP and RFP. For filopodia, fluorescence brightness was determined via the average maximum value of three perpendicular line scans. Background fluorescence was then subtracted from both membrane and filopodia intensities. On average, 100 filopodia from 25 cells were analyzed for each condition.

### Generation of polymer conformations

SAW models on a simple cubic lattice, with varied SAW step lengths and exclusions around an occupied site were used to model the IDP configurations. Such models serve as good simplistic descriptions of well-solvated polymers. They are especially amenable to the computation of entropy changes owing to their theoretical simplicity, and the ability to sample a large number of polymer conformations using relatively short Monte Carlo simulation simulations. A given step of a SAW on a 3D cubic lattice can be denoted as (PQR), where each of P, Q, and R can assume integer values. The allowed combinations are dictated by the choice of the SAW model deployed. In the simplest case of walks to adjacent (first nearest neighbor) sites can be denoted as the (001) models, which corresponds to allowed steps of “a” in any of the three coordinate directions, where “a” is the lattice constant. Further, the steric constraints between monomers can be approximated by excluding its neighbor sites from occupancy, up until the Xth nearest neighbor sites. The various SAW walks simulated are denoted thus as “nbrPQRexclX” (neighbor-PQR-excluded-X). The model “nbr112excl2” accordingly corresponds to 24 walk directions determined by the allowed combinations, as explained in the previous example. Moreover, for each lattice site occupied by a polymer bead, the neighboring lattice sites up to the second nearest neighbor (six first nearest neighbor sites + 12 s nearest neighbor sites) are excluded from occupancy. The choice of P, Q, R, and X determines the flexibility of the polymer chain. The set of SAW models used in the study are detailed in Supplementary Table 2. For each SAW model, the length of a step is 0.38 nm, the separation between successive *C*_α_ atoms in the protein backbone. For estimation of structural properties such as the radius of gyration (*R*_g_), the SAW polymers were allowed to move freely on the cubic lattice, while they were anchored onto the membrane substrate through a terminal bead for the calculations of entropy. The membrane substrate was modeled by simply excluding the relevant lattice sites from occupancy.

### Computation of chain entropy

The absolute entropy (S) from polymer chain conformations is calculated using the HSMC method developed by Meirovitch et al.^27^ Briefly, in the HSMC method, the reconstruction probability of a polymer chain conformation *i* (_*Pi*_^HSMC^) is obtained from the construction probabilities (*p*_*j*_) of each bond direction (*ν*_*j*_)

4 $$P_i^{{\mathrm{HSMC}}} = \mathop {\prod }\limits_{{\mathrm{j = 1}}}^N p_j\left( {\nu _j|\nu _{\left( {{\mathrm{j - 1}}} \right)}{\mathrm{, \ldots ,}}\nu _1} \right)$$

where *N* is the number of bonds in the polymer ((*N* + 1) is the number of beads). The *p*_*j*_ are measured using MC simulation of the entire future part of the chain (*ν*_*j*_, …, *ν*_*N*_) with the frozen past (*ν*_*(j-1)*_, …, *ν*_*1*_); *ν*_*j*_ representing a walk direction for the jth bond, which can range across the allowed walk directions for the given SAW model.

If *n*_MC_ is the number of MC steps deployed between the constructions of bonds (*j*−1) and *j*, and n_*j*_^*ν(k)*^ denotes the number of these steps in which a possible next bond direction ν_*j*_*(k)* is observed, then:

5 $$p_j\left( {\nu _j{\mathrm{(k)|}}\nu _{\left( {{\mathrm{j - 1}}} \right)}{\mathrm{, \ldots ,}}\nu _1} \right) = \frac{{n_j^{\nu \left( k \right)}}}{{n_{{\mathrm{MC}}}}}$$

Following the *n*_MC_ steps, the direction of the bond *j*, *ν*_*j*_ is chosen randomly, and the observed probability *p*_*j*_ is stored for the calculation of the reconstruction probability P_*i*_^HSMC^.

For accurate estimation of *p*_*j*_, *n*_MC_ for a given construction step is scaled with the length of the future chain segment. In our simulations, given the variety of SAW models used which differ in the allowed number of walk directions, *n*_MC_(*j*) is scaled as $\left( {\bar n{\mathrm{.}}n_{{\mathrm{nbr}}}.\left( {{n - j + 1}} \right)} \right)$, where $\bar n$ = 1000 and *n*_nbr_ is the number of allowed walk directions for the SAW model deployed. Between the *n*_MC_ steps corresponding to bonds *j* and (*j*+1), the segment (*ν*_*j*+1,_…,*ν*_*n*_) is reinitialized to an allowed extended conformation, and equilibrated for $\left( {\overline {n\prime } {\mathrm{.}}n_{{\mathrm{nbr}}}.\left( {{n - j + 1}} \right)} \right)$, steps where $\overline {n\prime }$=100.

The rigorous upper bound to the entropy (in units of *k*_B_, the Boltzmann constant) can be obtained from P_*i*_^HSMC^ using:

6 $$S^U{\mathrm{ = - }}\mathop {\sum}\limits_i {\frac{1}{{Z_{{\mathrm{SAW}}}}}{\mathrm{ln}}P_i^{{\mathrm{HMSC}}}}$$

where *Z*_SAW_ is the number of all possible SAWs. For a large number of sampled conformations (*N*_obs_), and since all the SAWs have identical statistical weight, *S*^U^ can be approximated by:

7 $$S^U \approx \frac{1}{{N_{{\mathrm{obs}}}}}\mathop {\sum}\limits_i {{\mathrm{ln}}P_i^{{\mathrm{HSMC}}}}$$

In addition, the lower bound to the entropy can be obtained as:

8 $$S^L{\mathrm{ = - }}\mathop {\sum}\limits_i {P_i^{{\mathrm{HSMC}}}{\mathrm{ln}}P_i^{{\mathrm{HSMC}}}}$$

9 $${\mathrm{ = - }}\frac{{\mathop {\sum }\nolimits_i \frac{1}{{Z_{{\mathrm{SAW}}}}}\left[ {P_i^{{\mathrm{HSMC}}}{\mathrm{ln}}P_i^{{\mathrm{HSMC}}}} \right]}}{{\mathop {\sum }\nolimits_i \frac{1}{{Z_{{\mathrm{SAW}}}}}P_i^{{\mathrm{HSMC}}}}}$$

10 $$\approx {\mathrm{ - }}\frac{{\mathop {\sum }\nolimits_i \frac{1}{{N_{{\mathrm{obs}}}}}\left[ {P_i^{{\mathrm{HSMC}}}{\mathrm{ln}}P_i^{{\mathrm{HSMC}}}} \right]}}{{\mathop {\sum }\nolimits_i \frac{1}{{N_{{\mathrm{obs}}}}}P_i^{{\mathrm{HSMC}}}}}$$

The resultant absolute entropy (*S*) is calculated as the mean of *S*^U^ and *S*^L^.

### Monte Carlo simulations

The structural properties for each SAW model and each length of polymer chain considered (*R*_g_) were calculated using 64 Monte Carlo simulation trajectories, with random number generator (Mersenne Twister) initialized using different seed values. Each of these simulations involved 10^7^ MC steps (a MC step is defined as a sequence of *N* local moves, chosen at random) divided over 10 blocks. The absolute entropies were calculated using either 96 or 120 generated reconstruction probabilities corresponding to different polymer conformations. A different value of seed for random number generator was used for each case.

### Conversion of entropy to relative curvature sensitivity

The Boltzmann distribution (Eq. 11) states that partitioning between bound and unbound proteins on vesicle surface (*K*_bind_) is equal to the exponent of the free energy difference between the free and bound protein (Δ*G*_bind_) divided by *k*_B_*T*. If curvature sensing is driven solely by entropy, then the enthalpy of binding (Δ*H*_bind_) would be constant among all vesicle diameters. Therefore, the relative partitioning between two vesicles with different diameters (*K*_bind,1_/*K*_bind,2_) can be calculated as shown in Eq. 12. The difference in binding entropy (Δ*S*_bind_) between the two vesicles is equal to the entropy change resulting from curvature (ΔΔ*S*_curvature_). Therefore, using 200 nm diameter SUVs as a reference, Eq. 14 was used to calculate the relative partitioning increase for simulated IDPs on vesicles in the 40 to 200 nm diameter range.

11 $$K_{{\mathrm{bind}}} = {\mathrm{exp}}\left[ { - \frac{{\Delta G_{{\mathrm{bind}}}}}{{k_BT}}} \right]{\mathrm{ = exp}}\left[ { - \frac{{\left( {\Delta H_{{\mathrm{bind}}}{\mathrm{ - T}}\left[ {\Delta S_{{\mathrm{bind}}}} \right]} \right)}}{{k_BT}}} \right]$$

12 $$\frac{{K_{{\mathrm{bind,1}}}}}{{K_{{\mathrm{bind,2}}}}} = {\mathrm{exp}}\left[ {\frac{{\left( {\Delta S_{{\mathrm{bind,1}}}{\mathrm{ - }}\Delta S_{{\mathrm{bind,2}}}} \right)}}{{k_B}}} \right] = {\mathrm{exp}}\left[ {\frac{{\Delta \Delta S_{{\mathrm{curvature}}}}}{{k_B}}} \right]$$

## Electronic supplementary material

Supplementary Information

## Data Availability

Data supporting the findings of this manuscript are available from the corresponding author upon reasonable request. The computer codes generated during this study are also available from the corresponding author upon reasonable request.
