# Supplementary material for: Synergy between intrinsically disordered domains and structured proteins amplifies membrane curvature sensing
Source: Nat Commun. 2018 Oct 8;9:4152. doi: 10.1038/s41467-018-06532-3 (PMC6175956; doi:10.1038/s41467-018-06532-3)
Supplement: Supplementary file 1 — Supplementary Information [file 41467_2018_6532_MOESM1_ESM.pdf]

Synergy between Intrinsically Disordered Domains and Structured Proteins Amplifies Membrane Curvature Sensing

Wade F. Zeno, Upayan Baul, Wilton T. Snead, Andre C.M. DeGroot, Liping Wang, Eileen M. Lafer, D. Thirumalai, and Jeanne C. Stachowiak

Supplementary Methods

FCS: Correcting τ_D to account for changes in solution viscosity Solution viscosity increases as salt concentration increases.[1] Therefore, it is necessary to adjust τ_D when the NaCl concentration is above or below the standard buffer value of 150 mM. Protein diffusivity (D) is related to viscosity (η) as shown in the Stokes-Einstein equation (Supplementary Equation 1).

$$D = \frac{k_B T}{6\pi\eta r_H} = \frac{\omega^2}{4\tau_D} \tag{1}$$

The relationship between diffusivity and diffusion time (τ_D) is also shown in Supplementary Equation 1. The parameters r_H and ω correspond to the protein hydrodynamic radius and confocal radius, respectively. With all other variables held constant, it is clear that η and τ_D are directly proportional. Therefore, τ_D was adjusted using the simple linear relationship shown in Supplementary Equation 2.

$$\tau_{D,2} = \frac{\eta_2}{\eta_1}\tau_{D,1} \tag{2}$$

Viscosity values of 0.89 cP, 0.90 cP, and 0.93 cP were used for NaCl concentrations of 10 mM, 150 mM, and 450 mM, respectively.[1] All diffusion times were adjusted to the equivalent diffusion time at 150 mM NaCl concentration.

FCS: Calibration of focal volume and determination of protein radii By rearranging Supplementary Equation 1, Supplementary Equation 3 is obtained, where r_H is directly proportional to τ_D with a slope of $2k_BT/3\pi\eta$. This slope corresponds to the conversion factor between τ_D and r_H when the NaCl concentration is 150 mM.

$$r_H = \frac{2k_B T}{3\pi\eta}\tau_D \tag{3}$$

The value of the slope was determined by experimentally measuring τ_D for GFP and transferrin, plotting the obtained τ_D values against their corresponding known r_H values, and performing a linear regression on the resulting plot, as shown in Supplementary Figure 12. Hydrodynamic radii of 2.3 nm and 3.7 nm were used for GFP[2] and transferrin[3], respectively. Once protein radii were determined, radii values were subsequently used to calculate projected protein area ($A = \pi r_H^2$) and membrane coverage.

Geometric Correction to B_{max} Due to the polymer-like nature of IDPs, r_p is expected to increase as membrane curvature decreases. The minimum possible r_p value corresponds to AP180CTD in solution, where highly water soluble IDP domains would be expected to occupy an approximately spherical shape. This same spherical shape would also be expected for an IDP domain tethered to a vesicle of zero diameter (infinite curvature). In contrast, the maximum possible r_p value should correspond to AP180CTD tethered to a flat membrane surface, where the protein would be expected to occupy an approximately hemispherical shape. Assuming that the polymer-like IDP

occupies a constant volume regardless of its shape (i.e. distribution of electrostatic and steric repulsion between amino acid residues remains constant), the volumes of the sphere (minimum r_p) and hemisphere (maximum r_p) should be equal, yielding $r_{p,2}/r_{p,1} = 2^{1/3}$, or approximately 1.25, Supplementary Figure 14a. Therefore r_p could vary from 6.0 nm, the value measured in solution, to 7.5 nm, depending on the curvature of the membrane surface. As displayed in Supplementary Figures 14b-c, using either value of r_p to adjust for the curvature dependence of B_{max} (Equation 2) largely removes the correlation between the corrected Bmax value and the vesicle radius. Specifically, the average corrected B_{max} value exhibited a <10% decrease, changing from 2.7 x 10^{-3} proteins nm^{-2} to 2.5 x 10^{-3} proteins nm^{-2} as r_p was increased from 6.0 to 7.5 nm, Supplementary Fig. 14b,c. This analysis confirms that the precise value of r_p, within its expected range, has minimal effect on the conclusions drawn regarding the geometric effect associated with saturated vesicles. Therefore, neglecting the curvature dependence of r_p does not significantly impact the interpretation of our data.

Supplementary Discussion

Comparison of curvature sensitivity at equal membrane coverage In crowded environments, the free energy change associated with protein binding to the membrane surface is reduced in magnitude as illustrated in Supplementary Equation 4. Here ΔG is the free energy associated with binding and ϕ is fractional coverage of the membrane surface by protein molecules, based on simple manipulations of expressions in the referenced text.[4] From this expression it is clear that the difference in free energy between crowded and dilute binding is a function of ϕ alone. Therefore, when two binding processes are compared under conditions of equal coverage, the impact of molecular crowding on each process is equivalent. Based on this reasoning we compared curvature sensitivity of different proteins under conditions for which average protein coverage on vesicles was equivalent (Figures 2e, 3b, 5c,f).

$$\left(\frac{\Delta G_{crowded}-\Delta G_{dilute}}{k_B T}\right)=-\ln(1-\phi) \qquad (4)$$

Exclusion of Membrane Entropy in Model To illustrate the validity of assuming that protein binding is unlikely to alter membrane curvature, consider the following comparison between the membrane bending energy and the change in the free energy per protein upon membrane binding. The computational model calculates the change in conformational entropy for a single membrane-bound IDP, associated with changes in membrane curvature. The largest predicted value of this change in entropy is approximately $\Delta S = -1.7k_B$ per protein as shown in the excerpt Supplementary Table 3 below. This value corresponds to the increase in conformational entropy associated with moving a membrane-bound IDP from a flat membrane surface to the surface of a 40 nm

diameter vesicle. Since the IDP of interest, AP180CTD, occupies a projected area on the membrane surface of approximately 100 nm^2, the change in entropy per area is 0.017 k_BT nm^{-2}. For comparison, the energy per membrane area required to deform a lipid membrane can be estimated using the Helfrich-Canham-Evans theory.[4] According to this theory, the energy required to transform a flat membrane disc into a hemispherical cap, G_{bend}, is approximately $4\pi K$, where K corresponds to the membrane's bending modulus – an intrinsic property of the membrane. Membranes used in our work were composed primarily of dioleoylphosphatidylcholine (DOPC). The bending modulus of DOPC membranes is approximately 20k_BT.[5] Therefore G_{bend} is approximately 250k_BT. As such, the energy per membrane area required to transform the 100 nm^2 patch of membrane beneath each protein into a hemisphere is approximately 2.5 k_BT nm^{-2}. From these first-order estimates it is clear that the energy per membrane area associated with the change in IDP conformation is at least two orders of magnitude less than the energy per membrane area associated with substantial membrane bending. Therefore, even if all of the energy associated with the change in IDP conformation could be coupled into membrane deformation, the membrane would still not be expected to deform significantly.

Supplementary Figures

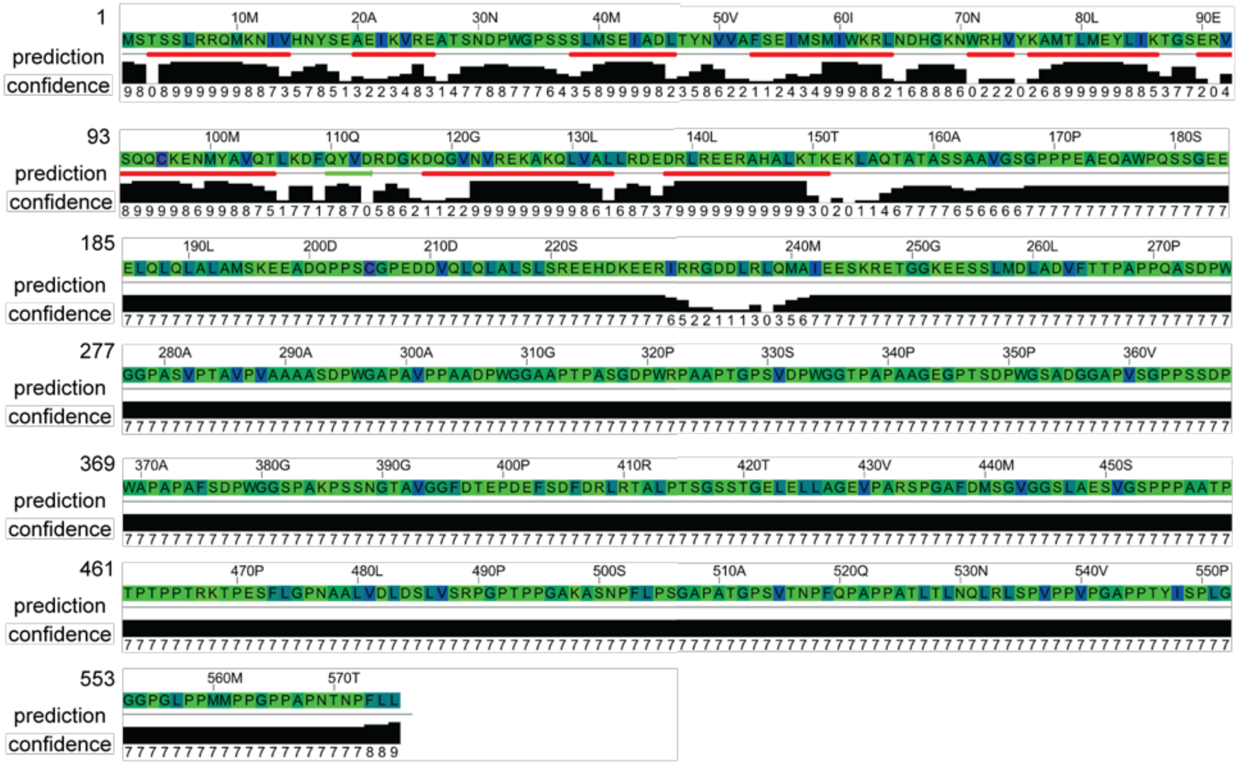

Supplementary Figure 1. Sequence structure predictions from JPred [6] full length Epsin1 (amino acids 1-575 of Rat Epsin1). Residues 1-164 correspond to the ENTH

domain, while residues 165-575 correspond to EpsinCTD. Results are represented as follows: (top row) the amino acid sequence, colored based on their predicted solvent accessibility – blue = buried and green = exposed; (middle row) predictions for helices shown as bold red lines and predictions for β-hairpins and strands shown as bold green arrows; (bottom row) the confidence index (0-9) of the prediction using both histograms and numbers - 9 corresponds to the highest confidence and 0 corresponds to the lowest confidence.

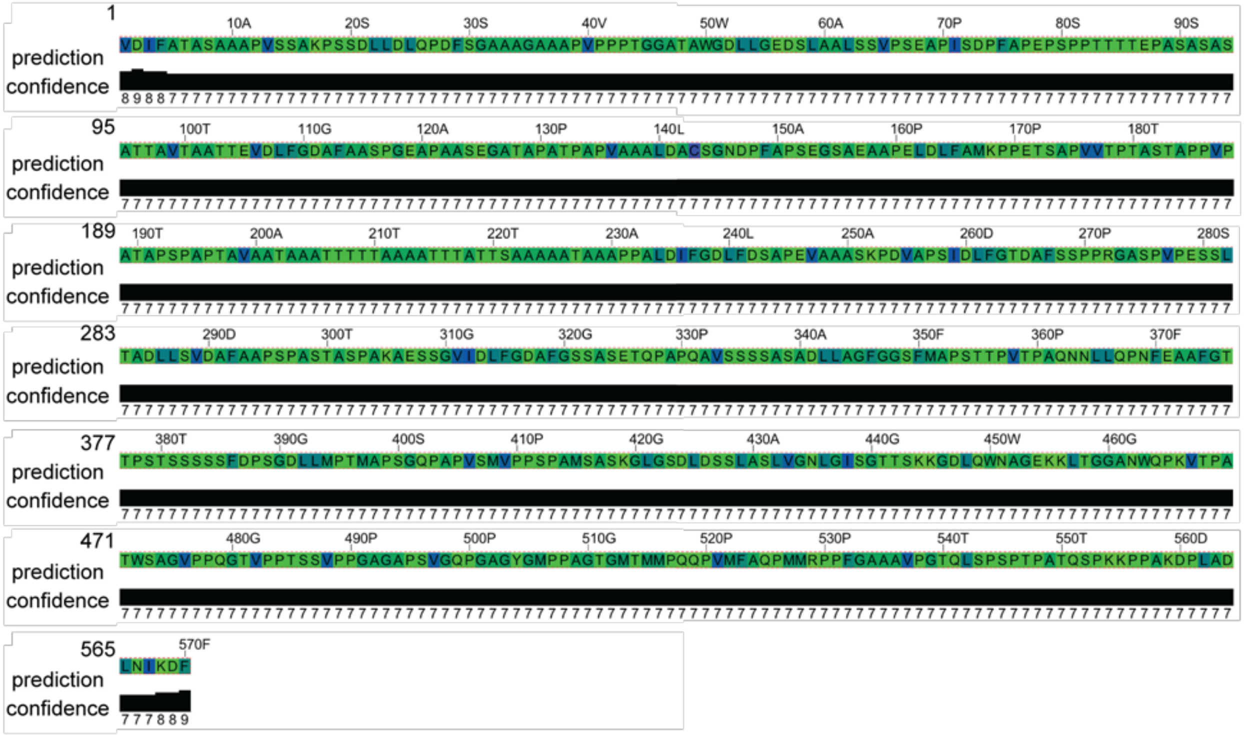

Supplementary Figure 2. Sequence structure predictions from JPred[6] for AP180CTD (amino acids 238-896 of Rat AP180). Results are represented as follows: (top row) the amino acid sequence, colored based on their predicted solvent accessibility – blue = buried and green = exposed; (middle row) predictions for helices shown as bold red lines and predictions for β-hairpins and strands shown as bold green arrows; (bottom row) the confidence index (0-9) of the prediction using both histograms and numbers - 9 corresponds to the highest confidence and 0 corresponds to the lowest confidence.

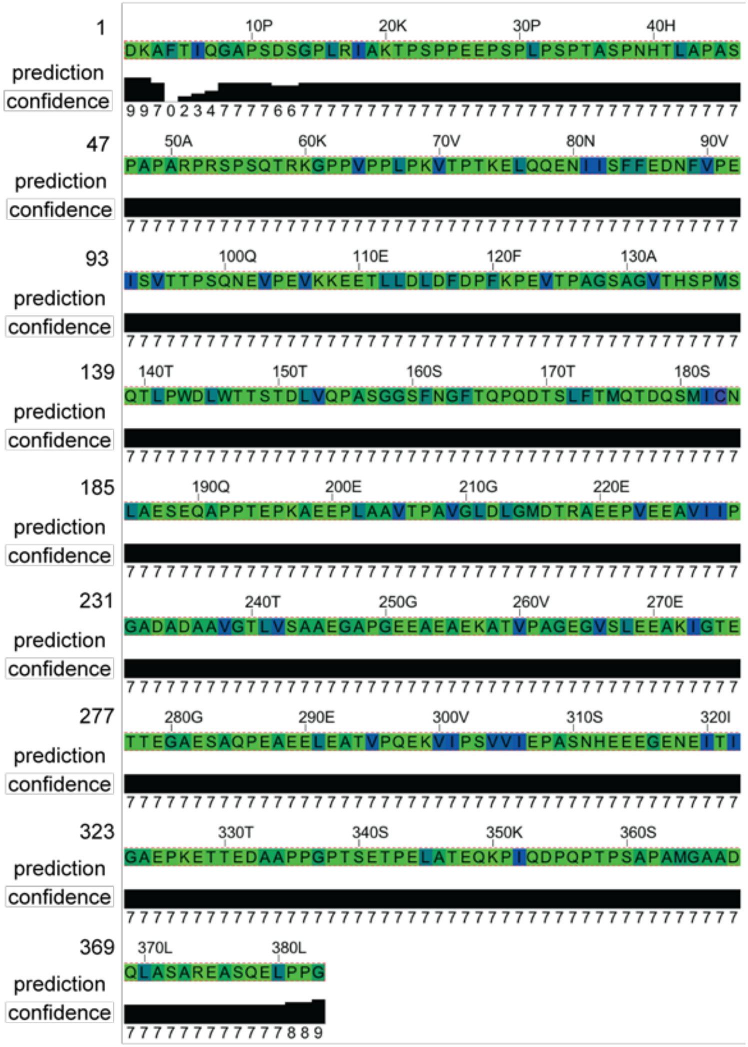

Supplementary Figure 3. Sequence structure predictions from JPred[6] for ΔAmphCTD (amino acids 240-622 of human Amphiphysin1, SH3 domain excluded). Results are represented as follows: (top row) the amino acid sequence, colored based on their predicted solvent accessibility – blue = buried and green = exposed; (middle row) predictions for helices shown as bold red lines and predictions for β-hairpins and strands shown as bold green arrows; (bottom row) the confidence index (0-9) of the prediction using both histograms and numbers - 9 corresponds to the highest confidence and 0 corresponds to the lowest confidence.

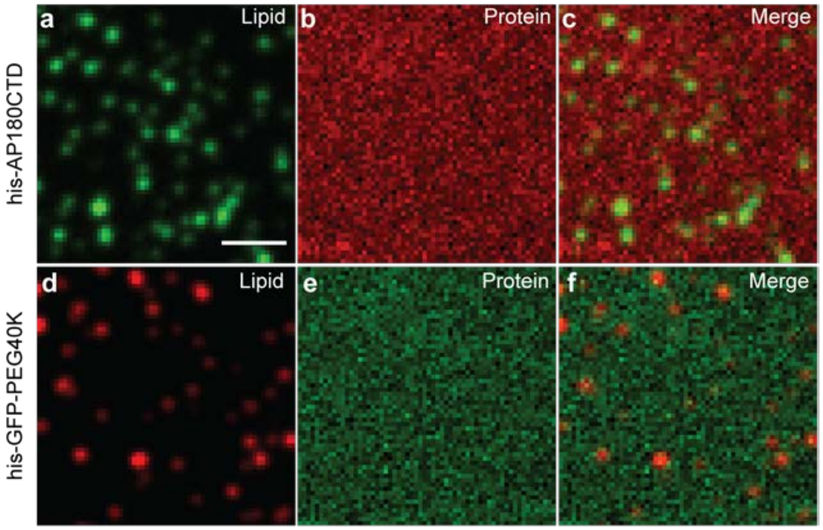

Supplementary Figure 4. His-AP180CTD and his-GFP-PEG40K did not bind measurably to vesicles when the necessary chelating agent, Ni^{2+}, was absent. Panels (a-c) depict fluorescence microscopy images corresponding to his-AP180CTD, while panels (d-f) correspond to his-GFP-PEG40K. SUVs used for his-AP180CTD experiments were composed of 80% DOPC, 16% DGS-NTA (lacking Ni^{2+}), 2% DP-EG10-biotin and 2% OG-DHPE. SUVs used for his-GFP-PEG40K were composed of 86% DOPC, 10% DGS-NTA (lacking Ni^{2+}), 2% DP-EG10-biotin, and 2% TR-DHPE. His-AP180CTD was labeled with ATTO-594 and present at a concentration of 200 nM. His-GFP-PEG40K was present at 100 nM and its fluorescence arose from the GFP portion of the molecule. Scale bar represents 2 microns.

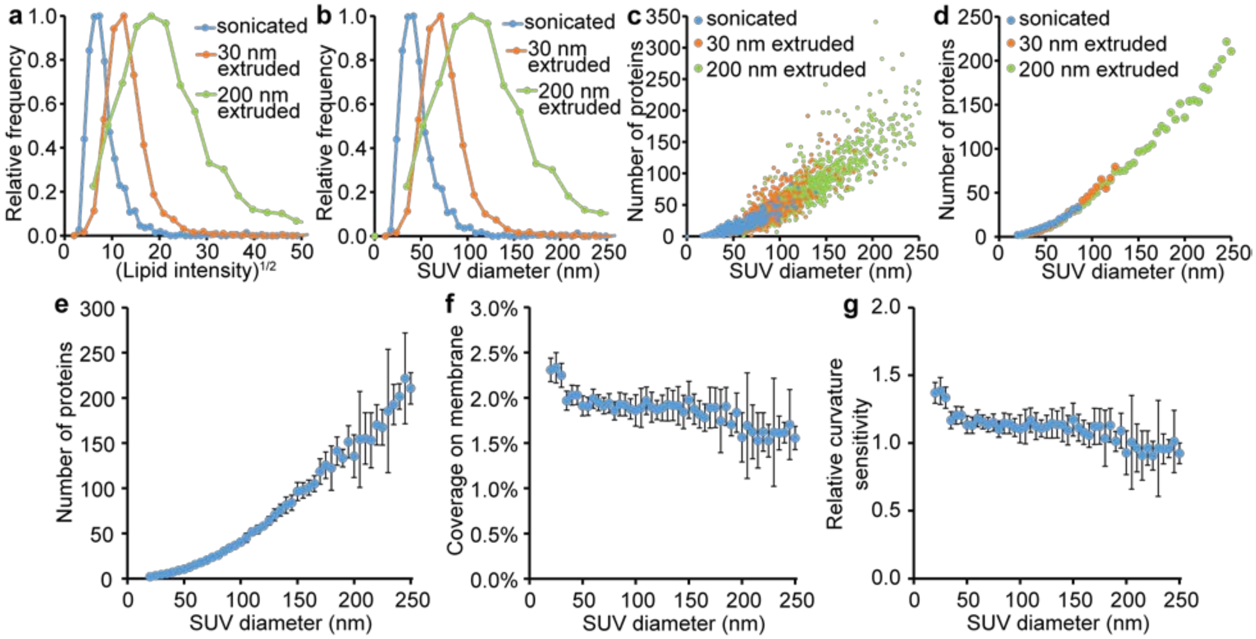

Supplementary Figure 5. Representative processing scheme for determining relative curvature sensitivity from the peak fluorescence values of protein and SUV puncta. Data shown here is for his-ΔENTH at 5 nM. **(a)** Distribution of (lipid channel peak fluorescence intensity)$^{1/2}$ for sonicated, 30 nm-extruded, and 200 nm-extruded SUVs. Lipid channel peak fluorescence intensity is proportional to vesicle surface area. Therefore its square root is proportional to vesicle diameter. **(b)** Conversion of (lipid channel peak fluorescence intensity)$^{1/2}$ distribution using a linear scaling factor of 5.69 nm (lipid intensity)$^{-1/2}$. This conversion factor was determined using a DLS measured mean of 71 nm for 30 nm-extruded SUVs. **(c)** Using the single protein calibration method from Supplementary Figure 9, and lipid size calibration from (**b**), the number of bound proteins per vesicle was plotted as a function of SUV diameter for all measured puncta. Sonicated, 30 nm-extruded, and 200 nm-extruded samples were overlaid. **(d)** A moving average of each vesicle preparation shows a distinct overlap of the diameter range covered by each preparation. **(e)** The data in (**c**) was concatenated and subjected to a moving average in 5 nm increments. **(f)** %Coverage was calculated by multiplying the number of proteins by the area of a single protein, and dividing that number by the surface area of the SUV. **(g)** Relative sensitivity was calculated via normalization of values in (**f**) using the average value of coverage for vesicles of 190-210 nm diameter. All error bars correspond to the 95% confidence interval of the mean within the 5 nm bins (N=27-343 for each bin and was acquired from 3 combined replicates).

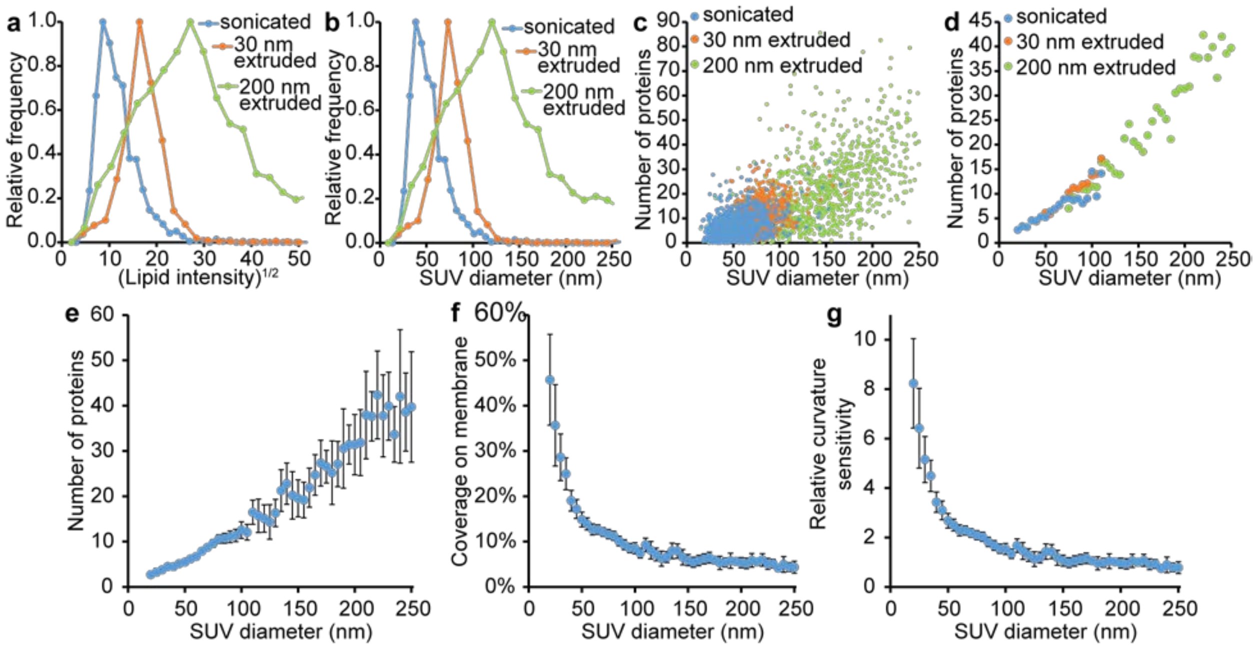

Supplementary Figure 6. Representative processing scheme for determining relative curvature sensitivity from the peak fluorescence values of protein and SUV puncta. Data shown here is for his-AP180CTD at 10 nM. **(a)** Distribution of (lipid channel peak fluorescence intensity)$^{1/2}$ for sonicated, 30 nm-extruded, and 200 nm-extruded SUVs. Lipid channel peak fluorescence intensity is proportional to vesicle surface area. Therefore its square root is proportional to vesicle diameter. **(b)** Conversion of (lipid channel peak fluorescence intensity)$^{1/2}$ distribution using a linear scaling factor of 4.45 nm (lipid intensity)$^{-1/2}$. This conversion factor was determined using a DLS measured mean of 73 nm for 30 nm-extruded SUVs. **(c)** Using the single protein calibration method from Supplementary Figure 9, and lipid size calibration from (**b**), the number of bound proteins per vesicle was plotted as a function of SUV diameter for all measured puncta. Sonicated, 30 nm-extruded, and 200 nm-extruded samples were overlaid. **(d)** A moving average of each vesicle preparation shows a distinct overlap of the diameter range covered by each preparation. **(e)** The data in (**c**) was concatenated and subjected to a moving average in 5 nm increments. **(f)** %Coverage was calculated by multiplying the number of proteins by the area of a single protein, and dividing that number by the surface area of the SUV. **(g)** Relative sensitivity was calculated via normalization of values in (**f**) using the average value of coverage for vesicles of 190-210 nm diameter. All error bars correspond to the 95% confidence interval of the mean within the 5 nm bins (N=16-259 for each bin and was acquired from 3 combined replicates).

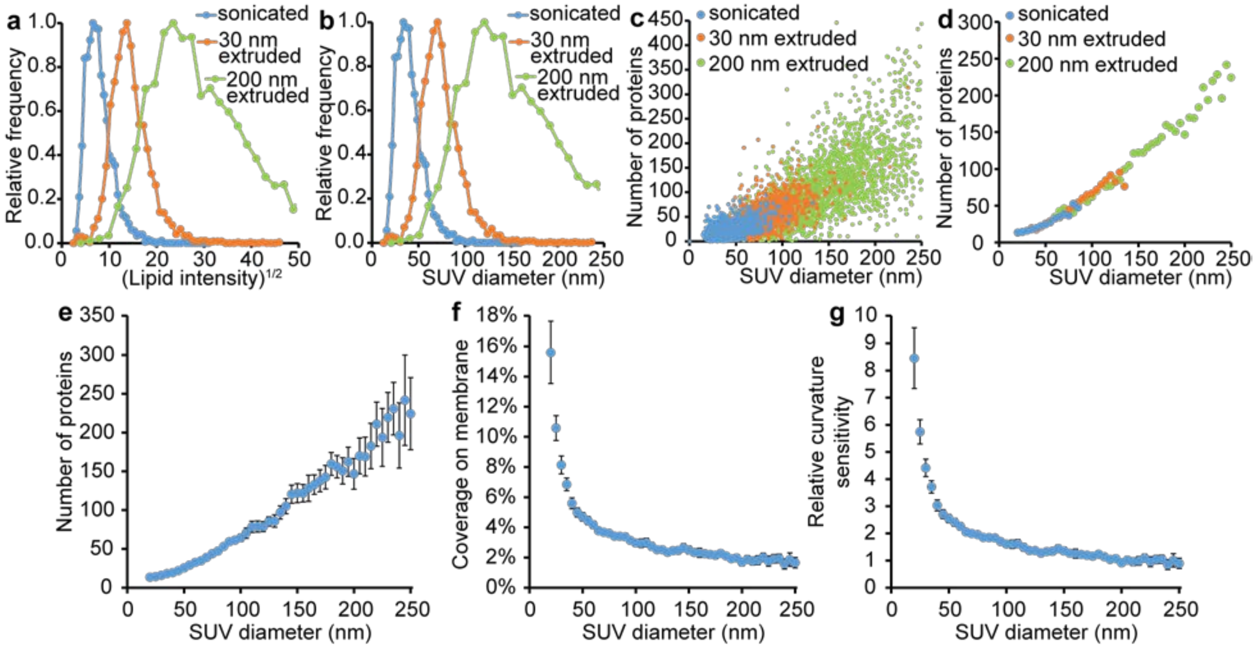

Supplementary Figure 7. Representative processing scheme for determining relative curvature sensitivity from the peak fluorescence values of protein and SUV puncta. Data shown here is for wt-ENTH at 450 nM. **(a)** Distribution of (lipid channel peak fluorescence intensity)$^{1/2}$ for sonicated, 30 nm-extruded, and 200 nm-extruded SUVs. Lipid channel peak fluorescence intensity is proportional to vesicle surface area. Therefore its square root is proportional to vesicle diameter. **(b)** Conversion of (lipid channel peak fluorescence intensity)$^{1/2}$ distribution using a linear scaling factor of 5.16 nm (lipid intensity)$^{-1/2}$. This conversion factor was determined using a DLS measured mean of 66 nm for 30 nm-extruded SUVs. **(c)** Using the single protein calibration method from Supplementary Figure 9, and lipid size calibration from (**b**), the number of bound proteins per vesicle was plotted as a function of SUV diameter for all measured puncta. Sonicated, 30 nm-extruded, and 200 nm-extruded samples were overlaid. **(d)** A moving average of each vesicle preparation shows a distinct overlap of the diameter range covered by each preparation. **(e)** The data in (**c**) was concatenated and subjected to a moving average in 5 nm increments. **(f)** %Coverage was calculated by multiplying the number of proteins by the area of a single protein, and dividing that number by the surface area of the SUV. **(g)** Relative sensitivity was calculated via normalization of values in (**f**) using the average value of coverage for vesicles of 190-210 nm diameter. All error bars correspond to the 95% confidence interval of the mean within the 5 nm bins (N=28-358 and was acquired from 3 combined replicates).

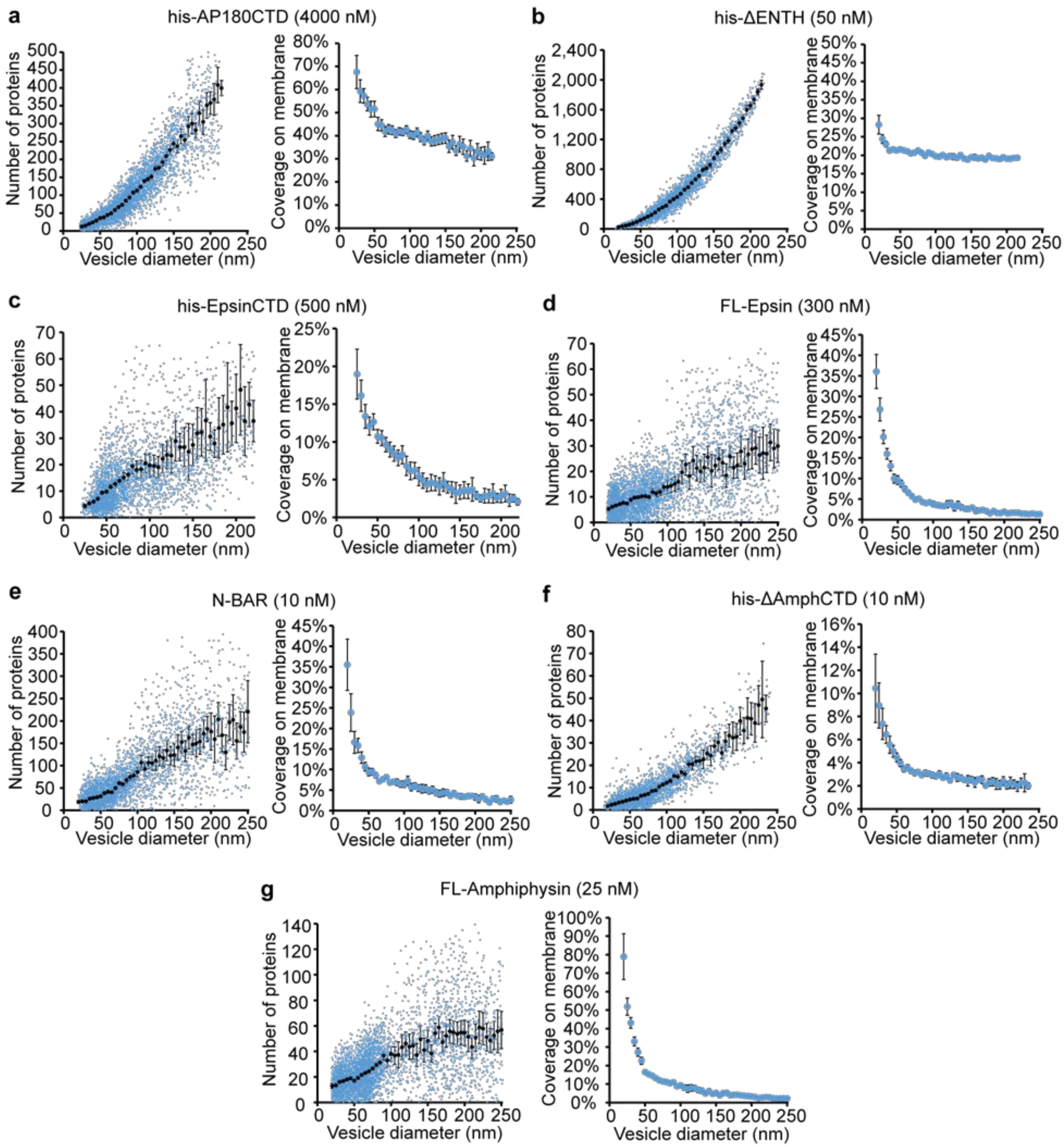

Supplementary Figure 8. Representative processing schemes for all proteins used in this work. Using the single protein calibration method from Supplementary Figure 9, and lipid size calibration method outlined in Supplementary Figures 5-7, the number of bound proteins per vesicle was plotted as a function of SUV diameter for all measured puncta, with the moving averages overlaid in 5 nm increments (left). %Coverage was then calculated by multiplying the number of proteins by the area of a single protein, and dividing that number by the surface area of the SUV (right). All error bars correspond to

the 95% confidence interval of the mean within the 5 nm bins (N=23-286 for **a**, 18-322 for **b**, 18-261 for **c**, 24-304 for **d**, 18-242 for **e**, 14-246 for **f**, 22-361 for **g**).

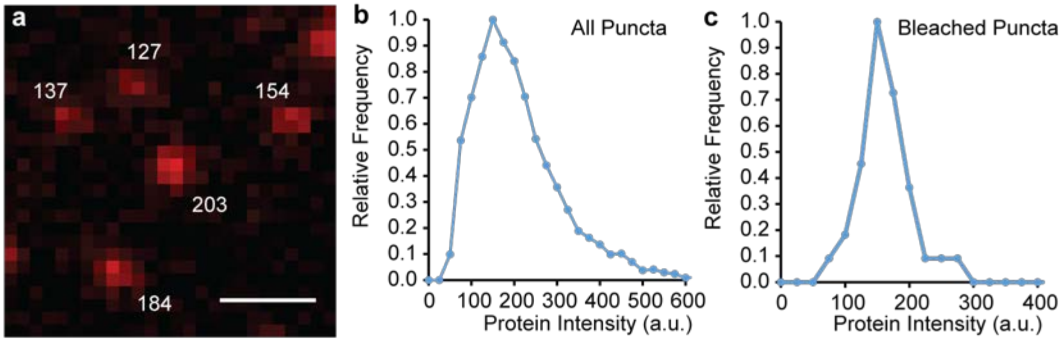

Supplementary Figure 9. Measuring the intensity of individual fluorescent dye-labeled proteins. **(a)** Representative image of ATTO-594 labeled proteins adhered passively to a coverslip surface. Scale bar is 1 μm long. The peak intensities of several puncta are labeled. The labeled protein is wt-ENTH, and the exposure time was 5 seconds. **(b)** Histogram of puncta intensities for images similar to (**a**). **(c)** Distribution of puncta intensity for single-proteins that exhibited single-step photobleaching within the imaging time course. The maximum peaks in **(b)** and **(c)** occur at similar intensities, indicating that the puncta used for calibration were single molecules.

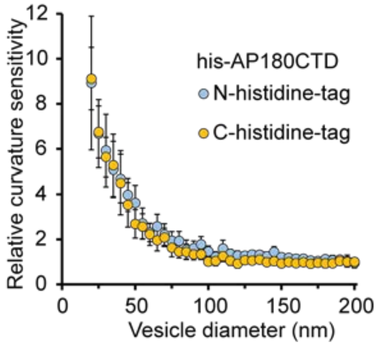

Supplementary Figure 10. Relative curvature sensitivities for N-terminally tagged and C-terminally tagged his-AP180CTD over a 20-200 nm SUV diameter range. Proteins were compared at concentrations that gave an average membrane coverage of approximately 3% (4 nM for N-tagged and 8 nM for C-tagged). SUVs were composed of 86% DOPC, 10% DGS-NTA, 2% DP-EG10-Biotin, and 2% OG-DHPE. Both proteins were labeled with ATTO-594. All error bars correspond to the 95% confidence interval of the mean within the 5 nm bins (N=20-184 for each bin and was acquired from 3 combined replicates).

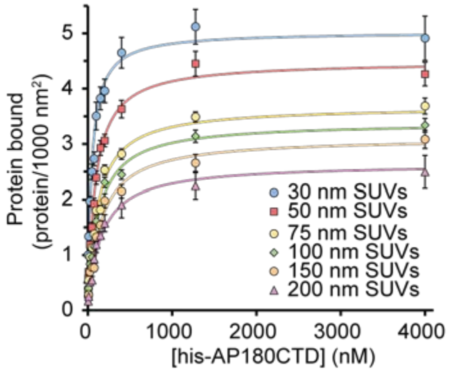

Supplementary Figure 11. Binding curves for his-AP180CTD on 30, 50, 75, 100, 150, and 200 nm diameter SUVs. Protein concentration was varied from 5 to 4000 nM. Solid lines represent the regression curves obtained from the Langmuir model in Equation 1. Error bars are represented by the 95% confidence interval of the mean (N=23-338 for each data point and was acquired from 3 combined replicates).

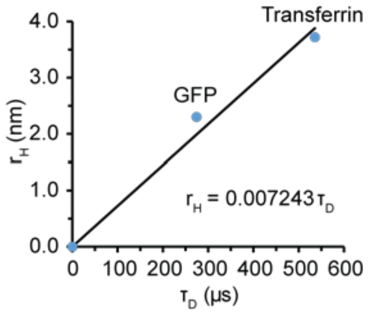

Supplementary Figure 12. FCS calibration with GFP and Transferrin for determining the conversion factor between diffusion time (τ_D) and hydrodynamic radius (r_H). Calibration was performed in 25 mM HEPES, 150 mM NaCl buffer (pH = 7.4).

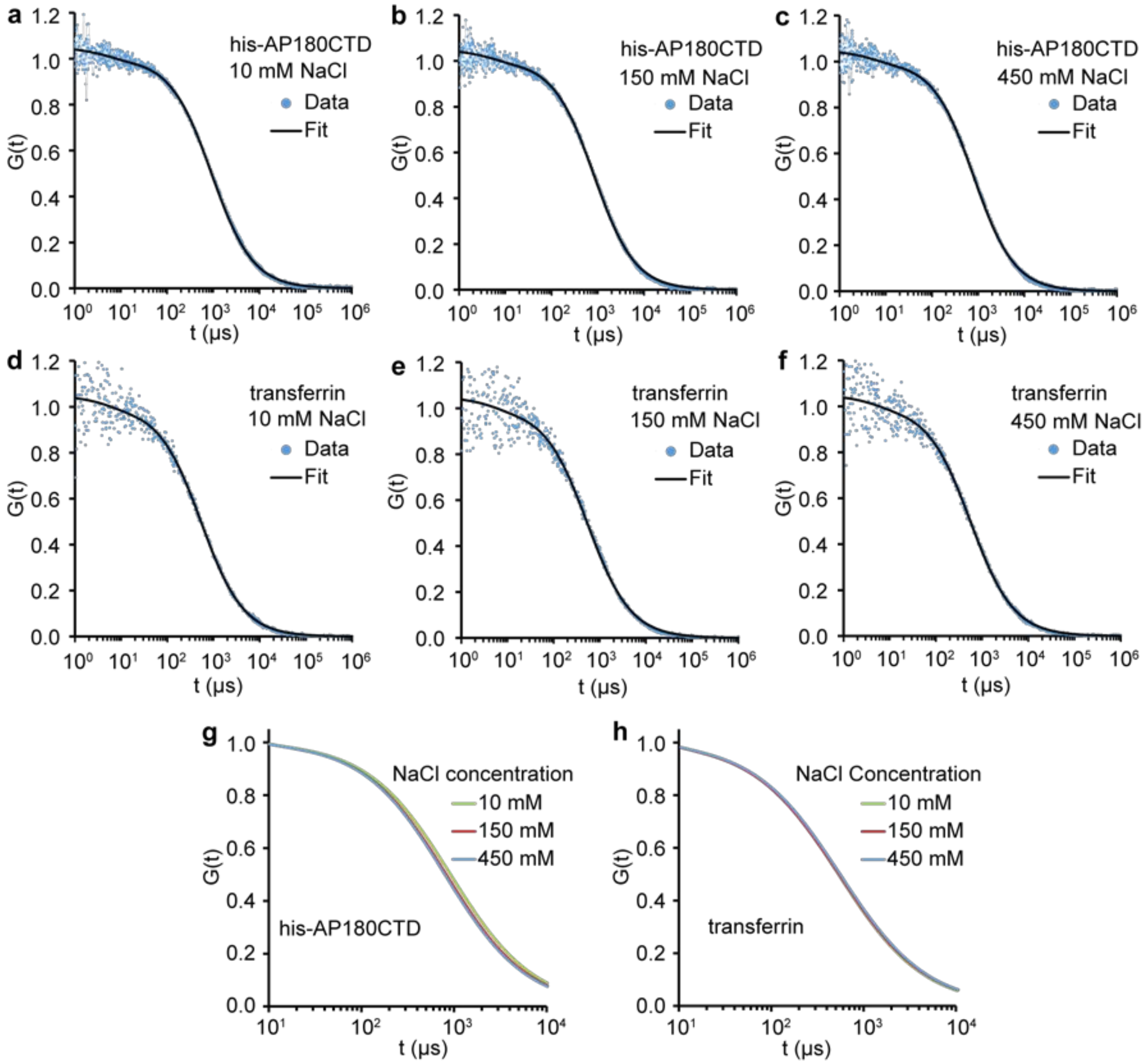

Supplementary Figure 13. Normalized FCS autocorrelation data and corresponding fits for his-AP180 (a-c) and transferrin (d-f) with NaCl concentrations of 10, 150, and 450 mM. Overlay of (g) his-AP180CTD and (h) transferrin autocorrelation function fits.

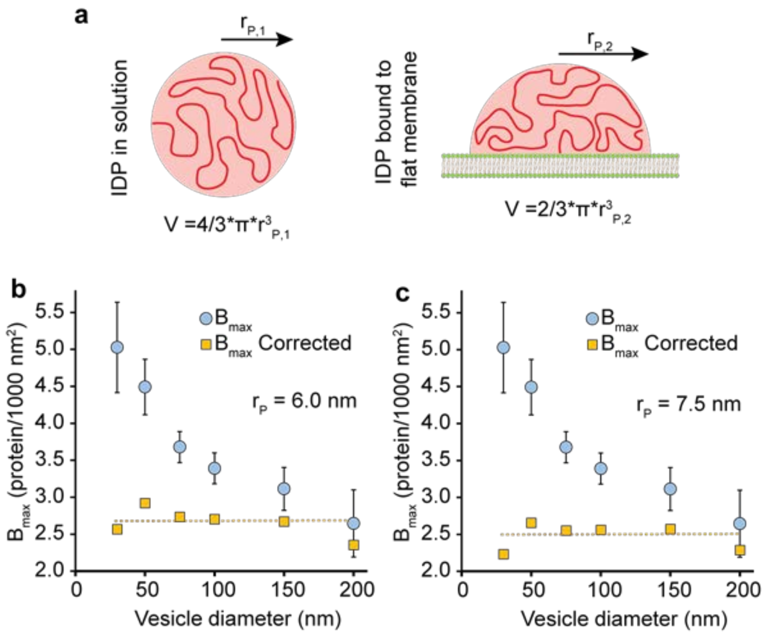

Supplementary Figure 14. Uncertainty about IDP radius does not significantly impact B_{max} corrections. (a) Depiction of an IDP in solution and tethered to a flat surface with the corresponding volumes occupied. Plots of geometric corrections for B_{max} when (b) r_p = 6.0 nm and (c) r_p = 7.5. (b) corresponds to AP180CTD's smallest possible radius and an average corrected B_{max} value of 2.7 x 10^{-3} proteins nm^{-2} while (c) corresponds to AP180CTD's largest possible radius and an average corrected B_{max} value of 2.5 x 10^{-3} proteins nm^{-2}. Error bars in **b** and **c** are represented by the standard deviation of 3 regression values obtained from fitting the mean, upper error, and lower error bounds in Figure 2a.

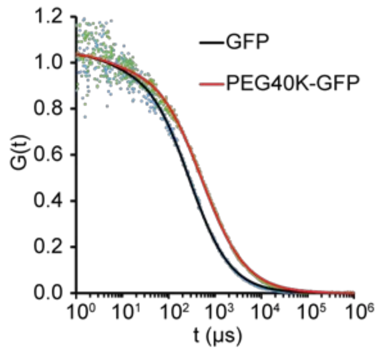

Supplementary Figure 15. Normalized FCS autocorrelation data and corresponding fits for GFP and PEG40K-GFP in 25 mM HEPES, 150 mM NaCl buffer (pH = 7.4).

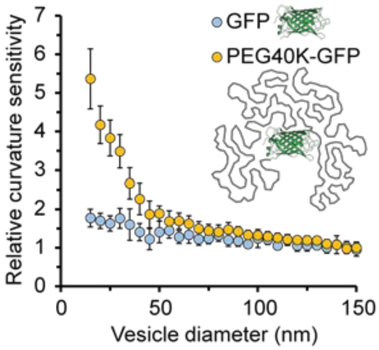

Supplementary Figure 16. Structures and relative curvature sensitivities for histidine tagged GFP and PEG40K-GFP over a 15-150 nm SUV diameter range. All proteins were compared at concentrations that gave an average membrane coverage of approximately 5% (25 nM for GFP and 75 nM for PEG40K-GFP). SUVs were composed of 86% DOPC, 10% DGS-NTA, 2% DP-EG10-Biotin, and 2% TR-DHPE. Error bars represent the 95% confidence interval of the mean for each 5 nm bin (N=19-176 for each bin and was acquired from 3 combined replicates).

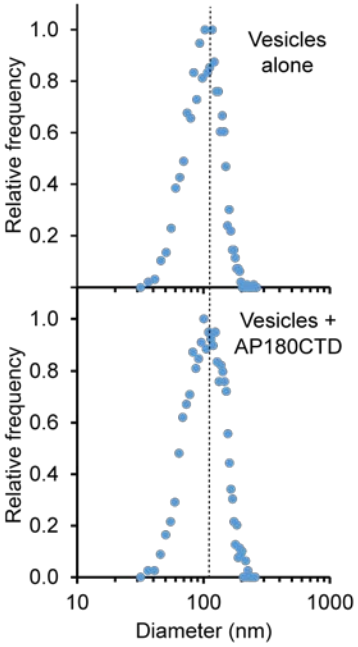

Supplementary Figure 17. The addition of IDPs to tethered vesicles does not substantially alter their size distribution. Size distribution of tethered vesicles in the absence and presence of his-AP180CTD. His-AP180CTD was labeled with ATTO-594 and incubated at a concentration of 20 nM. Vesicles were composed of 86% DOPC, 10% DGS-NTA, 2% DP-EG10-Biotin, 2% OG-DHPE and extruded at 100 nm.

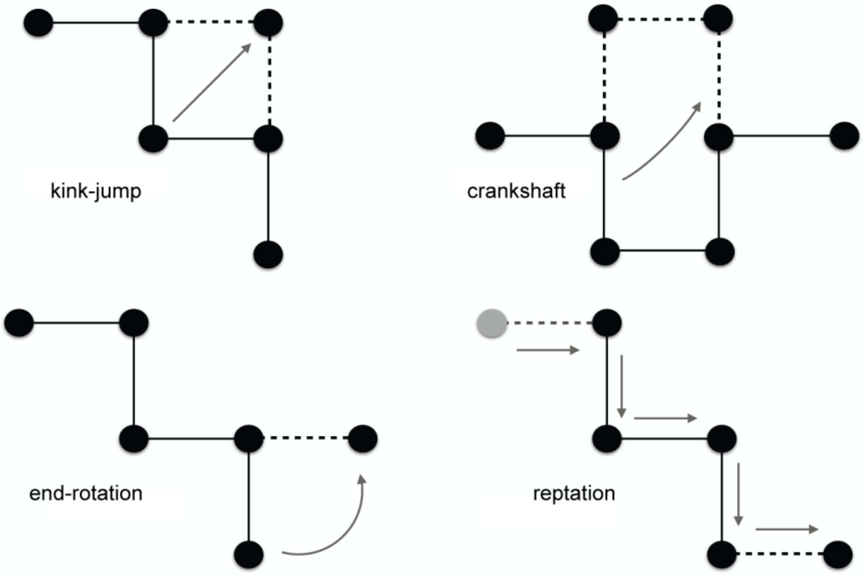

Supplementary Figure 18. Schematic representations of the various MC moves.

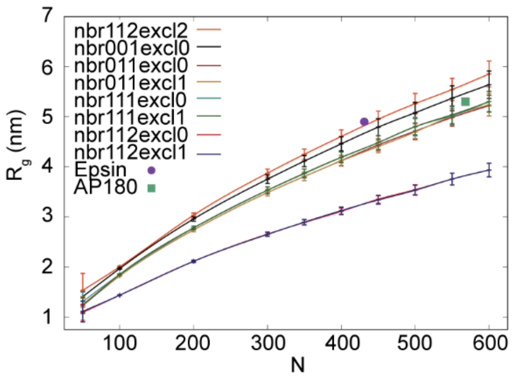

Supplementary Figure 19. R_g vs. N plots from the MC simulations with various SAW models. The experimental R_g values for AP180CTD and EpsinCTD are shown using a green square and a purple circle, respectively.[7]

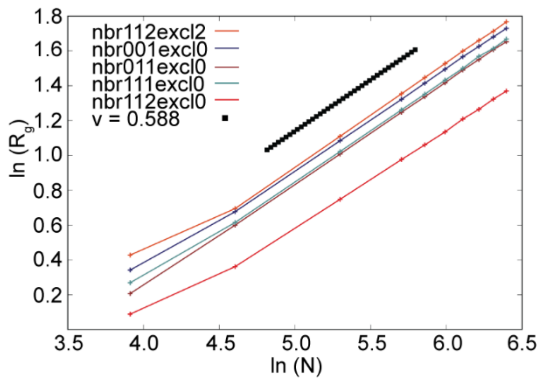

Supplementary Figure 20. R_g vs. N plots from the Monte Carlo simulations shown on a logarithmic scale. The expected scaling for random-coil like behavior[8] of SAW chains, with exponent 0.588, is shown with bold black squares for comparison ($R_g \sim N^v$, with v = 0.588 and N is the number of residues).

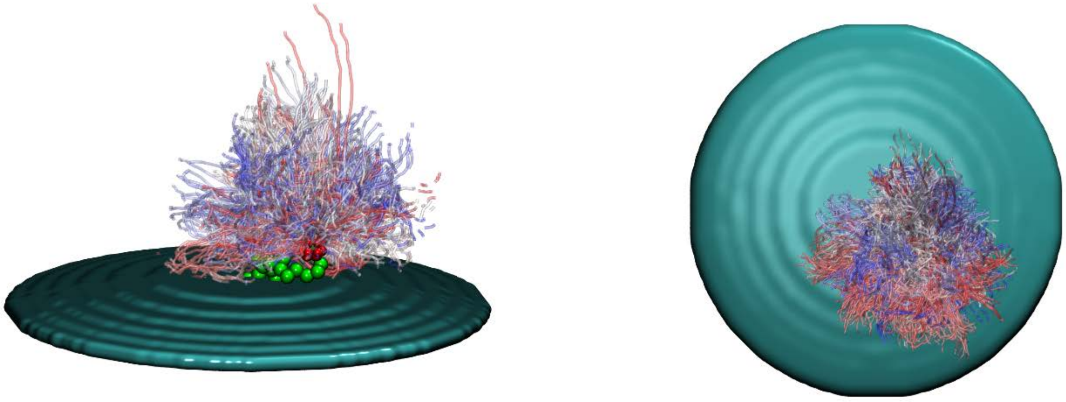

Supplementary Figure 21. Figures describing a bond construction step using the HSMC algorithm. (Left) Lateral view. The first 20 monomer units are shown with green spheres, and a fraction of the possible remaining segment conformations generated during the n_{MC} steps are shown with translucent tubes in a superposed representation (cross section view for clarity). Color of the tubes (red to blue) signify the arbitrary sequence in which the conformations were generated. Red beads denote possible locations of the next bead after the green beads. Some conformations may appear disconnected due to the cross-sectional view. (Right) Top view of the same set of conformations. In both figures, the tethering surface is shown in teal.

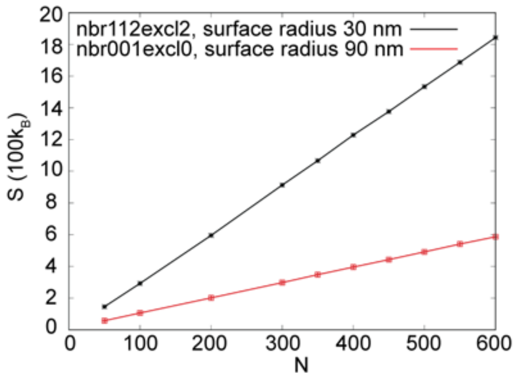

Supplementary Figure 22. The linear scaling of *S* with N, shown for two randomly chosen simulation environments. The small error bars signify good convergence obtained during the simulations.

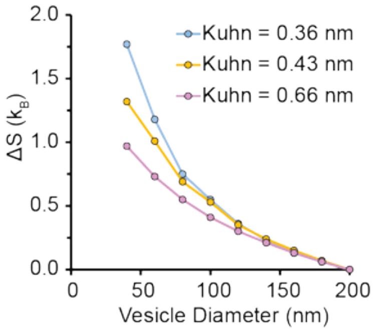

Supplementary Figure 23. Entropy change calculated using Equations 7-10 on spherical surfaces ranging in diameter from 40 to 200 nm. Kuhn length of the monomer segments was varied from 0.36 nm to 0.66 nm.

Supplementary Tables

Protein	NaCl Concentration	τ_D (μs)	N_p	α	η (cP)	adjusted τ_D (μs)
his-AP180CTD	10 nM	918 ± 19	3.2 ± 2.2	0.97 ± 0.02	0.89	929 ± 19
his-AP180CTD	150 nM	828 ± 17	2.2 ± 0.5	0.97 ± 0.02	0.90	828 ± 17
his-AP180CTD	450 nM	797 ± 17	2.0 ± 0.4	0.98 ± 0.02	0.93	771 ± 16
transferrin	10 nM	532 ± 17	0.91 ± 0.06	0.94 ± 0.01	0.89	539 ± 17
transferrin	150 nM	536 ± 21	0.58 ± 0.02	0.92 ± 0.01	0.90	536 ± 21
transferrin	450 nM	556 ± 15	0.67 ± 0.06	0.92 ± 0.03	0.93	538 ± 15
GFP	150 nM	274 ± 1	0.21 ± 0.01	0.96 ± 0.01	0.90	274 ± 1
PEG40K-GFP	150 nM	505 ± 6	0.30 ± 0.01	0.90 ± 0.01	0.90	505 ± 6

Supplementary Table 1 Regression values (τ_D, N_p, and α) for FCS fits and corresponding values of adjusted τ_D, which account for changes in viscosity (η) when salt concentrations deviate from 150 nM.

Model	# walk directions	# excluded neighbor sites
nbr001excl0	6	0
nbr111excl0	8	0
nbr111excl1	8	6
nbr011excl0	12	0
nbr011excl1	12	6
nbr011excl2	12	6+12
nbr112excl0	24	0
nbr112excl1	24	6
nbr112excl2	24	6+12

Supplementary Table 2 Number of walk directions and excluded neighbor sites for the various SAW models studied. For the excluded neighbor sites, their break-up into first and second nearest neighbor lattice sites is shown explicitly.

Diameter (nm)	ΔS (k_B) Flat Surface	Relative Curvature Sensitivity
40	1.65	5.2
80	1.02	2.7
100	0.86	2.4
140	0.57	1.8
160	0.48	1.6
200	0.33	1.4
300	0.18	1.2

Supplementary Table 3 Simulated relative curvature sensitivity for AP180CTD tethered to vesicles. ΔS represents the difference in configurational entropy for tethering on curved and planar surfaces. Values were obtained from the simulation described in Figure 3b for a Kuhn Length of 0.43 nm (nbr112excl2). Relative curvature sensitivity was calculated from ΔS using Equation 12.

Protein(s)	Primer Sequence (5’ to 3’)
Epsin-FL	Forward: GACTGAATTCATATGTCGACATCATCGCTGCGG<br>Reverse: GACTCTCGAGTTATAGGAGGAAGGGGTTAGT
AP180CTD-his (C-terminally tagged)	Forward: GATCGGATCCGTCGACATCTTTGCAACAGCA<br>Reverse: GATCGAATTCCTAATGGTGATGGTGATGGTGCAA GAAATCCTTGATGTTAAG
N-BAR	Forward: GACTGGATCCGCCGACATCAAGACGGGCATC<br>Reverse: GACTGAATTCCTAGGCCTTGTCGGCGTGCTGGTC
his-ΔAmphCTD	Forward: GACTGTCGACGACAAGGCCTTCACCATCCA<br>Reverse: GACTCTCGAGCTAGCCAGGAGGCAATTCCTGAG
TfR-GFP,<br>TfR-GFP-AP180CTD,<br>TfR-GFP-NfMCTD<br>(A206K site-directed mutagenesis)	Forward: GCACCCAGTCCAAACTGAGCAAAGA<br>Reverse: TCTTTGCTCAGTTTGGACTGGGTGC
NfMCTD-TfR-GFP	Forward: GCACATGAATTCATGAGCACATTTTCAGGAAGC<br>Reverse: GCACATGAATTCGTCACCCTGGGTGAC
TfR-RFP	Forward: GCACATACCGGTTATGGCCTCCTCCGAGGACGT<br>Reverse: GCACATGCGGCCGCTTAGGCGCCGGTGGAGT

Supplementary Table 4 Primer sequences used for the cloning DNA sequences in this work.

Supplementary References

1. Hai-Lang, Z. & Shi-Jun, H. Viscosity and density of water+ sodium chloride+ potassium chloride solutions at 298.15 K. *Journal of Chemical & Engineering Data* **41**, 516-520 (1996).
2. Hink, M. A. *et al.* Structural dynamics of green fluorescent protein alone and fused with a single chain Fv protein. *Journal of Biological Chemistry* **275**, 17556-17560 (2000).
3. Armstrong, J. K., Wenby, R. B., Meiselman, H. J. & Fisher, T. C. The hydrodynamic radii of macromolecules and their effect on red blood cell aggregation. *Biophysical journal* **87**, 4259-4270 (2004).
4. Phillips, R., Kondev, J., Theriot, J. & Garcia, H. *Physical biology of the cell*. (Garland Science, 2012).
5. Marsh, D. Elastic curvature constants of lipid monolayers and bilayers. *Chemistry and physics of lipids* **144**, 146-159 (2006).
6. Drozdetskiy, A., Cole, C., Procter, J. & Barton, G. J. JPred4: a protein secondary structure prediction server. *Nucleic acids research* **43**, W389-W394 (2015).
7. Busch, D. J. *et al.* Intrinsically disordered proteins drive membrane curvature. *Nat Commun* **6**, 7875, doi:10.1038/ncomms8875 (2015).
8. Doi, M. & Edwards, S. F. *The theory of polymer dynamics*. (Clarendon Press; Oxford University Press, 1987).
